# Repeat-Associated Fission Yeast-Like Regional Centromeres in the Ascomycetous Budding Yeast *Candida tropicalis*

**DOI:** 10.1371/journal.pgen.1005839

**Published:** 2016-02-04

**Authors:** Gautam Chatterjee, Sundar Ram Sankaranarayanan, Krishnendu Guin, Yogitha Thattikota, Sreedevi Padmanabhan, Rahul Siddharthan, Kaustuv Sanyal

**Affiliations:** 1 Molecular Mycology Laboratory, Molecular Biology and Genetics Unit, Jawaharlal Nehru Centre for Advanced Scientific Research, Jakkur, Bangalore, India; 2 The Institute of Mathematical Sciences, C.I.T. Campus, Taramani, Chennai, India; Duke University, UNITED STATES

## Abstract

The centromere, on which kinetochore proteins assemble, ensures precise chromosome segregation. Centromeres are largely specified by the histone H3 variant CENP-A (also known as Cse4 in yeasts). Structurally, centromere DNA sequences are highly diverse in nature. However, the evolutionary consequence of these structural diversities on *de novo* CENP-A chromatin formation remains elusive. Here, we report the identification of centromeres, as the binding sites of four evolutionarily conserved kinetochore proteins, in the human pathogenic budding yeast *Candida tropicalis*. Each of the seven centromeres comprises a 2 to 5 kb non-repetitive *mid* core flanked by 2 to 5 kb inverted repeats. The repeat-associated centromeres of *C*. *tropicalis* all share a high degree of sequence conservation with each other and are strikingly diverged from the unique and mostly non-repetitive centromeres of related *Candida* species—*Candida albicans*, *Candida dubliniensis*, and *Candida lusitaniae*. Using a plasmid-based assay, we further demonstrate that pericentric inverted repeats and the underlying DNA sequence provide a structural determinant in CENP-A recruitment in *C*. *tropicalis*, as opposed to epigenetically regulated CENP-A loading at centromeres in *C*. *albicans*. Thus, the centromere structure and its influence on *de novo* CENP-A recruitment has been significantly rewired in closely related *Candida* species. Strikingly, the centromere structural properties along with role of pericentric repeats in *de novo* CENP-A loading in *C*. *tropicalis* are more reminiscent to those of the distantly related fission yeast *Schizosaccharomyces pombe*. Taken together, we demonstrate, for the first time, fission yeast-like repeat-associated centromeres in an ascomycetous budding yeast.

## Author Summary

Centromeres aid in high fidelity chromosome segregation. Paradoxically, centromere DNA sequences are rapidly evolving in fungi, plants, and animals. Centromere DNA sequences in fungi can be unique in each chromosome or share conserved features such as motifs for sequence specific protein binding, pericentric repeats, or transposon-rich elements. Ascomycetous fungi, in particular, show a wide range of diversity in centromere sequence elements. However, no ascomycetous budding yeast species is known to possess repeat-associated centromeres in all of its chromosomes. Here, we identified and mapped all seven centromeres in an ascomycete, a rapidly emerging human pathogenic yeast, *Candida tropicalis*. The repeat-associated centromeres of highly homogeneous DNA sequences in *C*. *tropicalis* are significantly diverged from the mostly non-repetitive unique centromeric DNA sequences of its closely related sequenced species, *Candida albicans*, *Candida dubliniensis* and *Candida lusitaniae*. Structurally, the centromeres of *C*. *tropicalis* more closely resemble those of the distantly related fission yeast *Schizosaccharomyces pombe*. Thus, we discover rapidly diverging repeat-associated centromeres in an ascomycetous budding yeast and provide evidence of emergence of repeat-associated centromeres via two independent evolutionary events in ascomycetous fungi.

## Introduction

The high fidelity segregation of replicated chromosomes to daughter cells during cell division is essential in maintaining genome integrity. It is achieved by a dynamic and well-coordinated kinetochore-microtubule interaction on a specialized chromosomal element, known as the centromere. Strikingly, the centromere DNA shows rapid diversification in its sequence, length, and the organization of sequence elements across different species [1–3]. The centromere has been categorized into point and regional primarily based on its length. In addition, there are kinetochore protein complexes which are associated specifically to either the point or regional centromere [4]. Point centromeres, which are typically <400 bp long with conserved DNA elements (*CDE*s) but lacking DNA sequence repeats, appear to have evolved only once and are restricted to the *Saccharomyces* lineage [4]. However, the centromeres of most other organisms are regional in nature and span from as small as a few tens of kilobases (kb) as in fission yeast *Schizosaccharomyces pombe* to as large as multiple megabases (Mb) in length as observed in plants and animals. The large regional centromeres of most plants (reviewed in [5, 6]) and animals (reviewed in [7]) are composed of an array of either repetitive sequences or transposable elements. A classic example is the human centromeres that are organized as 171 bp monomeric repeats arranged into a higher ordered alpha satellite sequence (reviewed in [8]). The regional centromeres of two ascomycetous fungi, *Neurospora crassa* and *S*. *pombe*, and a basidiomycetous fungus *Cryptococcus neoformans* are much shorter (40 to 300 kb in length) and composed of either transposon-rich repetitive sequences as in *N*. *crassa* [9, 10] and in *C*. *neoformans* [11], or a heterogeneous central core sequence (*cnt*) flanked by two distinct inverted repeats (*imr* and *otr*) that are conserved across the centromeres in *S*. *pombe* [12–14]. It is noteworthy that the repeat-associated fungal centromeres lack tandem arrays of repeats as observed in the centromeres of higher metazoans. Interestingly, centromeres of chicken [18], potato [19] and unicellular red alga *Cyanidioshyzon merolae* [20] represent a distinct class where both repetitive and repeat-less centromeres exist in the same genome. On the other hand, shorter small regional centromeres of 3 to 5 kb non-repetitive, unique sequences have been identified in three *Candida* species–*Candida albicans* [15], *Candida dubliniensis* [16] and *Candida lusitaniae* [17]. Interestingly, the centromeres in these organisms lack any sequence conservation shared among different chromosomes in the same species. However, *CEN1*, *CEN5*, and *CENR* in *C*. *albicans* as well as in *C*. *dubliniensis* possess pericentric inverted repeats which are unique to each centromere [16]. The driving force enabling the evolution of centromeres with such remarkable diversity both in the DNA sequence as well as structure, rather than a common optimized centromere configuration, across eukaryotes remains an enigma [1].

The centromere DNA sequence and the organization of the sequence elements are rapidly evolving even in closely related species of three major forms of eukaryotic life—fungi, plants, and animals [1, 18]. In addition, a series of events including–(a) neocentromere formation [19–24] by centromere repositioning at ectopic sites with no obvious DNA sequence homology to the native centromere, (b) selective inactivation of a centromere in a dicentric chromosome [25–28], and (c) the presence of identical sequences elsewhere in the genome that do not serve as centromere/neocentromere sites in various organisms support the conclusion that centromere specification is largely epigenetically regulated (reviewed in [29, 30]).

The centromere specific histone H3 variant CENP-A (also known as Cse4 in yeasts) [31] is considered to be an epigenetic hallmark of active centromeres [32]. The unique structure of CENP-A chromatin provides the foundation to recruit other kinetochore proteins belonging to the Constitutive Centromere Associated Network (CCAN), Ndc80 complex and Dam1/ Ska complex [33], and nucleates kinetochore assembly in most organisms [34]. However, the mechanism(s) of CENP-A loading at a particular locus across species required for centromere specification and its propagation in subsequent generations remains unclear. As shown in *S*. *pombe*, CENP-A loading at the centromere is probably regulated via distinct processes leading to the establishment and propagation of a centromere in most organisms [35]. *De novo* CENP-A recruitment without any pre-existing mark is crucial to establish a centromere, whereas loading of CENP-A molecules during every cell cycle is important for the propagation of already established centromeres [36].

A common feature of the large regional centromeres in ascomycetous fungi is their inherent association with DNA repeats. Detailed studies on the centromeres of *S*. *pombe* revealed that centromere associated repeats provide structural determinants in *de novo* CENP-A recruitment [37]. In contrast, studies in the human pathogenic budding yeast *C*. *albicans*, which possesses small regional centromeres [3] reveal that the centromere DNA sequence (*CEN7*), that lacks pericentric repeats, fails to form functional centromere *de novo* on a naked plasmid harboring the *CEN7* because CENP-A could not be recruited to the plasmid *CEN7* [38]. This result implies that centromeres are epigenetically specified in absence of the pericentric repeats in *C*. *albicans* [38]. However, it remains to be tested whether centromeres with inverted repeats (such as *CEN5*) can recruit CENP-A *de-novo* in *C*. *albicans*.

*Candida* species, the most commonly encountered human fungal pathogens, cause a wide variety of mucosal infections and organ invasion in immunocompromised patients [39]. Although *C*. *albicans* has been long known to be the most abundant *Candida* species isolated from patients, recent global surveillance programs suggest that non-*albicans Candida* (NAC) species are rapidly emerging as a serious threat due to widespread use of antifungal drugs [40, 41]. In particular, infections caused by *Candida tropicalis*, a parasexual human pathogenic yeast, has been increased dramatically worldwide. Particularly in sub-tropical regions of Asia-Pacific, the number of patients with *C*. *tropicalis* infection is higher than that caused by *C*. *albicans* [40, 42]. Earlier, we reported centromere properties of *C*. *albicans* [15] and *C*. *dubliniensis* [16]. Here, we report the identification of the centromeres as binding sites of four evolutionarily conserved kinetochore proteins in *C*. *tropicalis*, which has a 30 Mb sequenced diploid genome arranged into 23 supercontigs [43]. A comparative analysis of centromeres suggests a rapid divergence not only in the centromere DNA sequence but also in the organization of the sequence elements in these closely related *Candida* species. Interestingly, pericentric repeats are shown to be important for *de novo* CENP-A recruitment on *C*. *tropicalis* centromeres. Based on the striking structural resemblance of centromeres and the necessity of pericentric repeats for *de novo* centromere formation both in *C*. *tropicalis* and *S*. *pombe*, we propose an independent evolution of repeat-associated centromeres in budding and fission yeasts.

## Results

### Kinetochore proteins are well conserved in *C*. *tropicalis*

**Fig 1:**
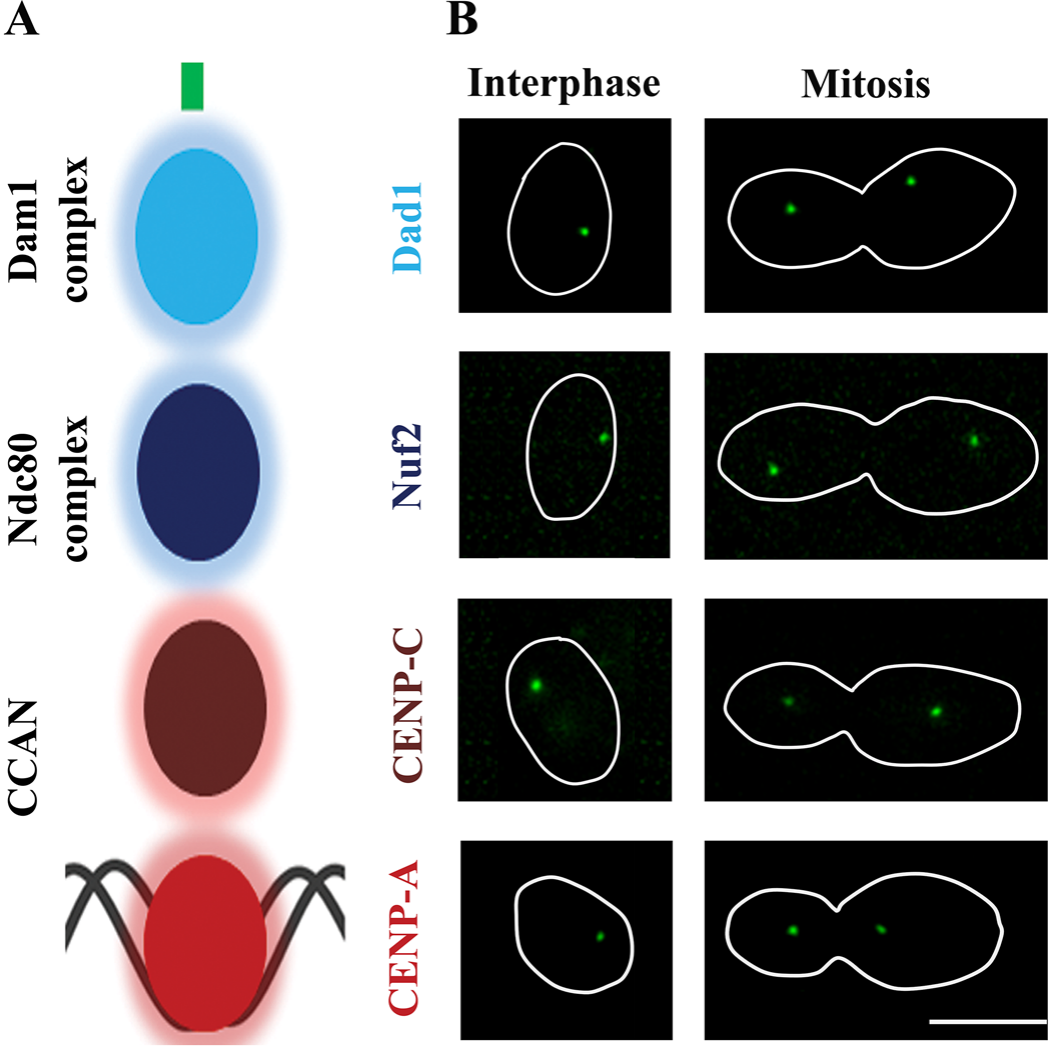
Identification of kinetochore proteins in *C*. *tropicalis*. (A) An illustration showing the kinetochore organization in yeasts. (B) Live cell fluorescence microscopic images of indicated proteins at two different stages of the cell cycle: interphase (unbudded) and mitotic (large-budded). Scale bar, 5 μm.

We identified four putative kinetochore proteins in *C*. *tropicalis*- CtCENP-A (Cse4), CtCENP-C (Mif2), CtNuf2 and CtDad1 (Fig 1A). Each of these proteins shares a high degree of sequence conservation to those of the closely related species *C*. *albicans* (S1A Fig). Subcellular localization of these proteins in *C*. *tropicalis* revealed localization patterns typical of kinetochore proteins in related yeasts [44–47]: a single punctate structure representing clustered kinetochores in unbudded G1 cells that then segregated into two puncta in large-budded cells undergoing mitosis (Fig 1B). In addition, indirect immunofluorescence microscopy with anti-Cse4 antibodies [45], which are specific to CtCENP-A (S1B Fig), and anti-tubulin antibodies revealed CENP-A to be localized near the spindle pole bodies (S1C Fig). On the basis of the sequence similarities and localization patterns at two different stages of the cell cycle, we conclude that these genes encode conserved kinetochore proteins in *C*. *tropicalis*.

### Kinetochore proteins are essential for chromosome segregation during mitosis in *C*. *tropicalis*

**Fig 2:**
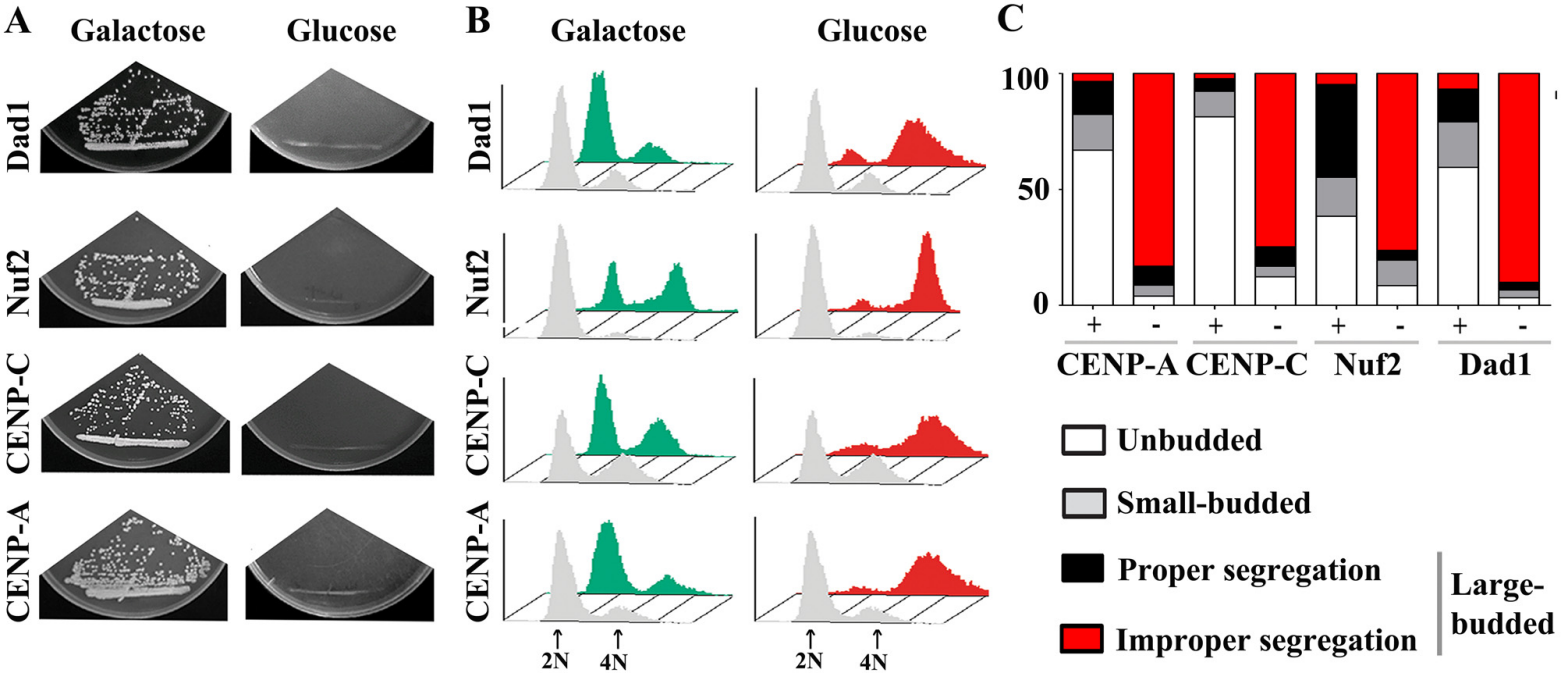
Depletion of conserved kinetochore proteins causes chromosome mis-segregation in *C*. *tropicalis*. (A) CENP-A, CENP-C, Nuf2 and Dad1 are essential for viability in *C*. *tropicalis*. *C*. *tropicalis* conditional mutant strains expressing the only copy of the above mentioned genes under the *GAL1* promoter were streaked on plates with galactose (permissive) or glucose (restrictive) as the sole carbon source and were photographed after 2 to 3 days of incubation at 30°C. The *GAL1* promoter is induced in the presence of galactose but repressed in glucose containing media in *C*. *tropicalis*. (B) FACS analysis of the conditional mutant strains of CENP-A, CENP-C, Nuf2 and Dad1 grown in either permissive (galactose), or non-permissive (glucose) media. The *x*-axis and *y*-axis represent the DNA content and number of cells respectively. (C) The distribution of unbudded, small-budded and large-budded (G2/M stage) cells of indicated mutant strains grown in either permissive (+) or non-permissive (-) conditions. The nuclear morphology was visualized by DAPI staining after 6 h of growth in permissive or non-permissive condition and the cells exhibiting proper or improper chromosome segregation during the G2/M stage are counted (n = >250 cells). The *y*-axis represents the percentage cell population.

Kinetochore proteins are important for chromosome segregation in eukaryotes and their depletion results in chromosome segregation defects due to improper microtubule-kinetochore interactions, which may lead to cell cycle arrest due to activation of the spindle assembly checkpoint. For conditional expression of genes, we identified the *GAL1* promoter sequence in *C*. *tropicalis* (See Methods). To test the function of these putative kinetochore proteins on chromosome segregation in this diploid organism, one copy of each gene was replaced by a marker gene and the remaining copy was placed under the control of the *GAL1* promoter. The inability of the conditional mutant strains to grow under non-permissive conditions confirmed that each of these four kinetochore proteins is essential for viability in *C*. *tropicalis* (Fig 2A). Moreover, flow cytometry (FACS) analysis revealed an accumulation of large budded cells at the G2/M stage during growth in non-permissive conditions (Fig 2B and S2 Fig). A significant number of the arrested cells had either an unsegregated nuclear mass at the bud neck, or unequally segregated nuclei indicating an arrest due to mitotic checkpoint activation (Fig 2C and S2 Fig). Taken together, these results strongly suggest that each of these proteins is essential for proper chromosome segregation in *C*. *tropicalis*.

### Genome-wide mapping reveals seven unique but overlapping CENP-A- and CENP-C- rich regions in *C*. *tropicalis*

**Fig 3:**
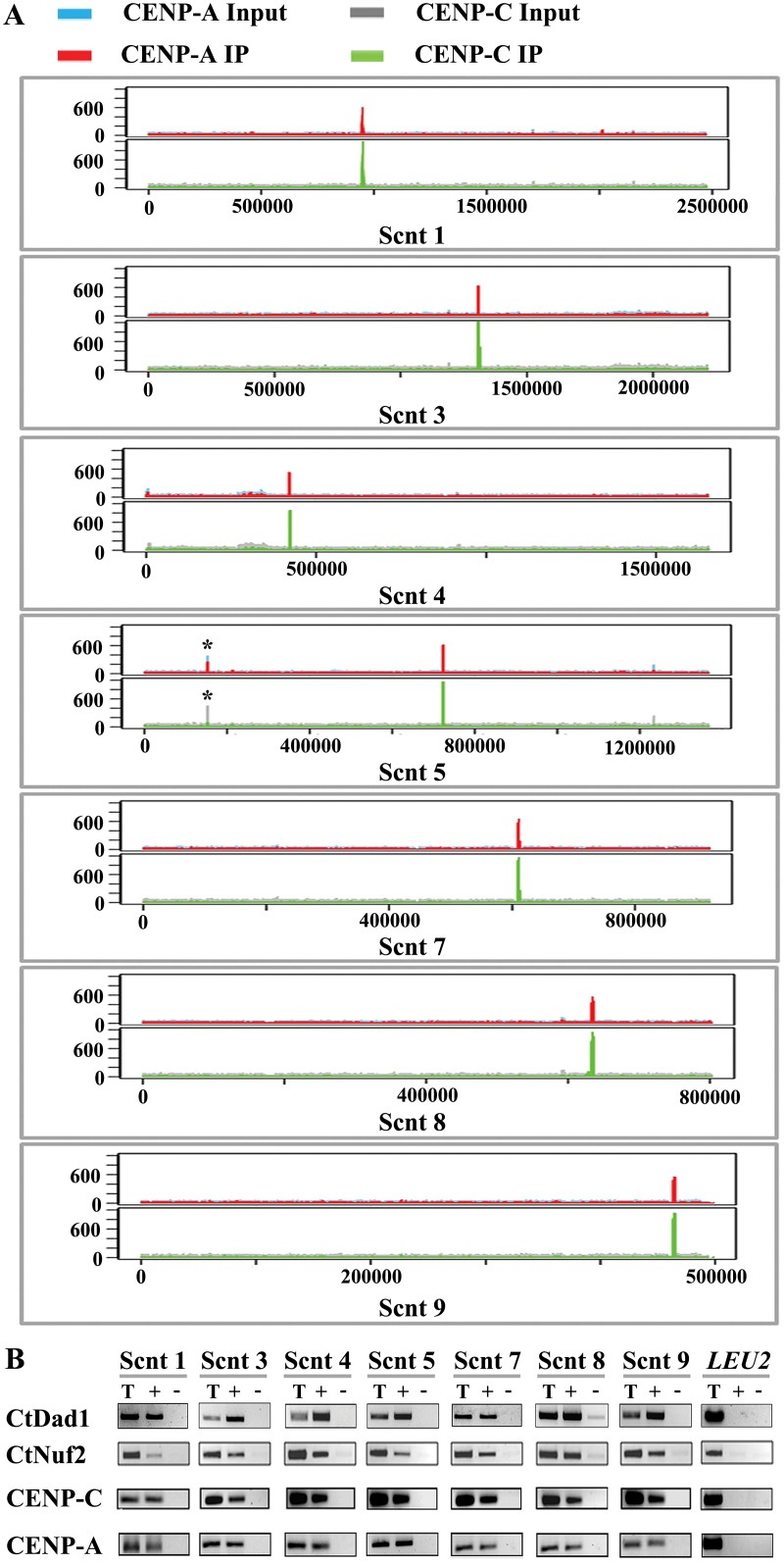
CENP-A and CENP-C ChIP-seq analyses identified seven centromeres in *C*. *tropicalis*. (A) CENP-A and CENP-C ChIP-seq reads along the seven enriched supercontigs are shown. Here, *x*-axis and *y*-axis represent the coordinates of the chromosomal regions and the distribution of sequence reads of the specific supercontig respectively. The asterisk (*) denotes the peak observed in input library. (B) Enrichment of indicated proteins at the centromeres on different supercontigs. ChIP DNA fractions of the indicated proteins were analyzed by PCR using a primer-pair unique to each supercontig (see S4 Table for primer sequences). Ct*LEU2*, a non-centromeric locus, was used as negative control. ‘T’, Total DNA, ‘+’, IP DNA with antibodies and ‘-’, beads only control.

Having identified authentic kinetochore proteins, we next sought to map the centromeres in the *C*. *tropicalis* genome as the binding sites of CENP-A and CENP-C by chromatin immunoprecipitation followed by next generation sequencing (ChIP-seq) [48]. The sequenced *C*. *tropicalis* strain MYA-3404 (*CSE4*/ *CSE4*) and its derivatives CtKS201 (*MIF2*/ *MIF2*-TAP) were used for CENP-A (anti-Cse4 antibodies) and CENP-C (anti-Protein A antibodies) ChIP experiments respectively. Analysis of the ChIP-seq reads against the *C*. *tropicalis* genome [43] identified seven CENP-A- and CENP-C-bound overlapping but unique regions as centromeres in *C*. *tropicalis* (Fig 3A and S3A and S3B Fig). Primers designed from the seven unique enriched regions identified by ChIP-seq were used to validate the enrichment of CENP-A and CENP-C binding by analyzing ChIP DNA of each of the four kinetochore proteins namely, CENP-A, CENP-C, Nuf2, and Dad1 on seven supercontigs as compared to a non-centromeric locus (Ct*LEU2*) using semi-quantitative PCR assays (Fig 3B). Moreover, each of these seven regions resides within a long ORF-free region (S1 Table), a common centromeric feature observed in most organisms.

To determine chromosomal identity of each of these centromeres, the chromosomes of *C*. *tropicalis* (MYA-3404) were first separated on CHEF gels (see methods). The probes were PCR amplified from a unique region adjacent to each centromere. The specific signals on the Southern blot of the CHEF gels revealed that at least five regions reside on different chromosomes (S4 Fig). Due to limited resolution of higher molecular weight chromosomes, the chromosomal identity of two regions (Scnt 3 and Scnt 4) could not be unambiguously verified by the CHEF analysis. These analyses, together with the previously reported 14 telomeric-linked scaffold ends [43], strongly suggest that there are seven pairs of chromosomes in *C*. *tropicalis*.

### Kinetochore proteins bind specifically to 2 to 3 kb regions on each chromosome

**Fig 4:**
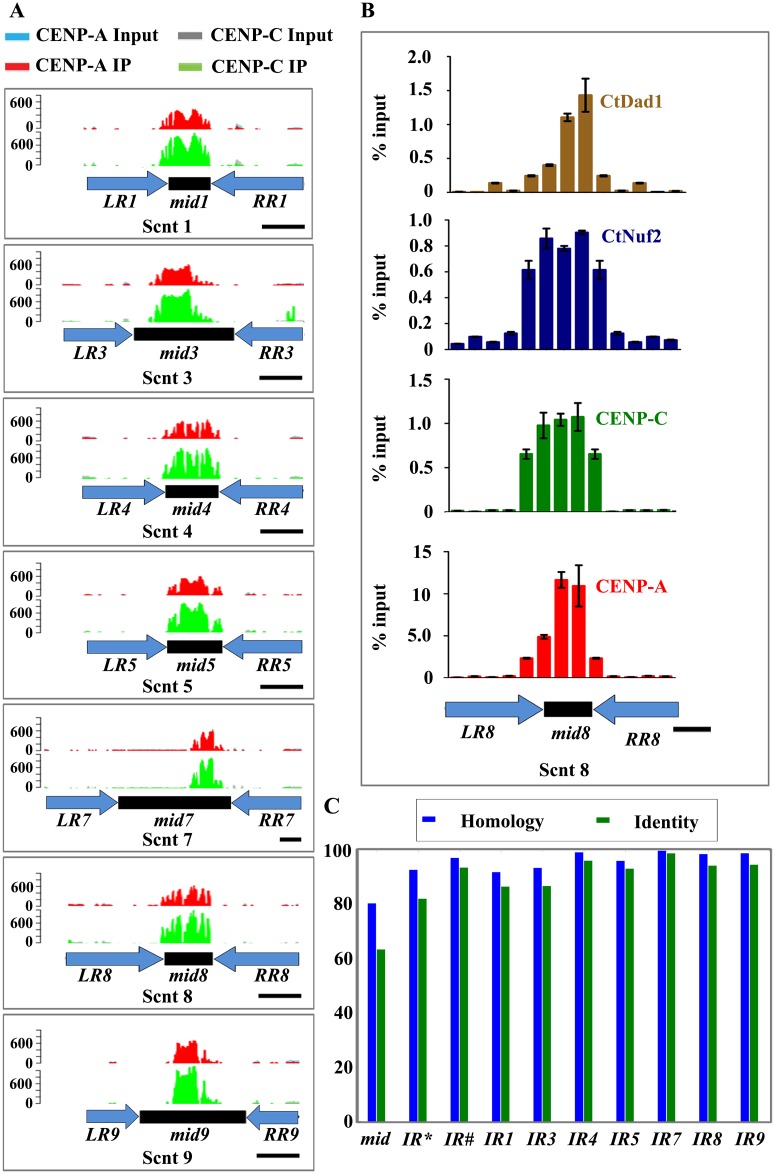
Repeat-associated centromere organization in *C*. *tropicalis*. (A) ChIP-seq analysis revealed that CENP-A and CENP-C bind to the *mid* core region in *C*. *tropicalis*. Here, the *x*-axis represents the structural components of a centromere in *C*. *tropicalis* and the *y*-axis represents the distribution of sequence reads of CENP-A (red) or CENP-C (green) ChIP DNA of the respective supercontig. Schematic representations of structural components of a centromere in *C*. *tropicalis* are shown below each ChIP-seq reads. Black boxes represent the *mid* regions, blue arrows indicate inverted repeats, the left repeat (*LR*) and the right repeat (*RR*). Scale bar, 2 kb. (B) ChIP-qPCR assays confirm the binding of kinetochore proteins across the centromere in Scnt 8. The *x*-axis represents coordinates on the supercontigs and the *y*-axis denotes the qPCR value as a percentage of the total chromatin input with standard error mean (SEM). Scale bar, 2 kb (C) Sequence conservation between the *mid* core regions (*mid*s) and inverted repeats (*IR*s) from different centromeres in *C*. *tropicalis*. Homology is calculated as the percentage of aligned nucleotides in a pair-wise alignment, measured from the shorter sequence. On the other hand, identity is the percentage of aligned and conserved nucleotides in the pair-wise alignment, again measured from the shorter sequence. Averages are calculated from all pair-wise alignments, weighted by length. IR* and *IR*# denote average value with respect to either others or self respectively.

ChIP-seq analyses show a complete overlap in binding of CENP-A and CENP-C to a 2 to 3 kb region on each of the seven centromeres (Fig 4A and S1 Table). To validate the length of the CENP-A/CENP-C binding regions obtained by the ChIP-seq analysis, we scanned the enrichment of each of the four above mentioned kinetochore proteins on the ORF-free region of Scnt 8 by ChIP followed by quantitative PCR (ChIP-qPCR) with primers designed at approximately 1 kb intervals across the 10 kb region of Scnt 8. This analysis revealed that these kinetochore proteins were enriched over a 3 kb (632950–636200) region on Scnt 8 (Fig 4B) and confirms the results obtained from the ChIP-seq experiment. Binding of evolutionarily conserved kinetochore proteins on the same locus proves that the region on each of the seven chromosomes is an important part of a functional centromere in *C*. *tropicalis*.

### A dramatic divergence in centromere sequence and its organization in related *Candida* species

The sequence analysis of centromeric DNA revealed that all seven centromeres in *C*. *tropicalis* have common structural elements comprising a non-repetitive *mid* core flanked by inverted repeats (*IR*s) (Fig 4A and S2 Table). The average length of the *mid* core region is 3.5 kb and is flanked by *IR*s of an average length of each repeat of 3.5 kb. This is a dramatic transition in the centromere organization in comparison with the centromeres of other closely related *Candida* species, *C*. *albicans* [15, 49], *C*. *dubliniensis* [16] and *C*. *lusitaniae* [17]. Incidentally, *CEN1*, *CEN5* and *CENR* of *C*. *albicans* and *C*. *dubliniensis* also contain non-conserved short pericentric repeats. The binding of both CENP-A and CENP-C is restricted to 2 to 3 kb non-repetitive *mid* core region in all centromeres in *C*. *tropicalis* (Fig 4A). A similar length of CENP-A binding (3 to 5 kb) has been observed in *C*. *albicans* [15], *C*. *dubliniensis* [16], and *C*. *lusitaniae* [17] suggesting a striking conservation in the length of CENP-A chromatin that provides the platform for kinetochore formation [50–52]. On the other hand, the AT-content of the CENP-A-bound *mid* core regions is found to be 64% in *C*. *tropicalis*, which is marginally less than the overall AT-content of the genome (67%). A similar AT-content of CENP-A bound centromere DNA (65%) was observed in *C*. *albicans* [15]. Thus, in spite of the observed rapid change in the centromere DNA sequence and its organization, these closely related species employ a similar length and composition (in terms of the AT-content) of the centromere DNA for the recruitment of kinetochore proteins.

### The centromeres of *C*. *tropicalis* possess highly homogenized inverted repeats (*IR*s) around a non-repetitive *mid* core

*In silico* analysis of centromere sequences in *C*. *tropicalis* revealed that the inverted repeats (*IR*s) and *mid* core regions share a high degree of sequence homology across the centromeres (Fig 4C and S5A–S5C Fig). Between different chromosomes, the *mid* core regions share 80% homology and 63% identity while the *IR*s show an average of 92% homology and 82% identity. However, the conservation is much higher between the left and right repeats (*LR* and *RR*) of the same centromere, with an average of 97% homology and 93% identity (Fig 4C and S5D Fig). In addition, we also observed that tandem direct repeats are present within each inverted repeat (S5E Fig and S3 Table). These groups of tandem repeats were prominent across all arms (except Scnt 9, which lacks the final group). However, the copy number varied significantly among arms (S3 Table). The observed high level of sequence conservation of the *mid* core and inverted repeats among the different centromeres in *C*. *tropicalis* suggests that these regions might have undergone homogenization via intra- and inter-chromosomal recombinatorial events. Such process may be facilitated by the close association of centromeres in the clustered kinetochores of *C*. *tropicalis* (Fig 1B).

### Chromosomal rearrangement involving centromeric regions in closely related *Candida* species

**Fig 5:**
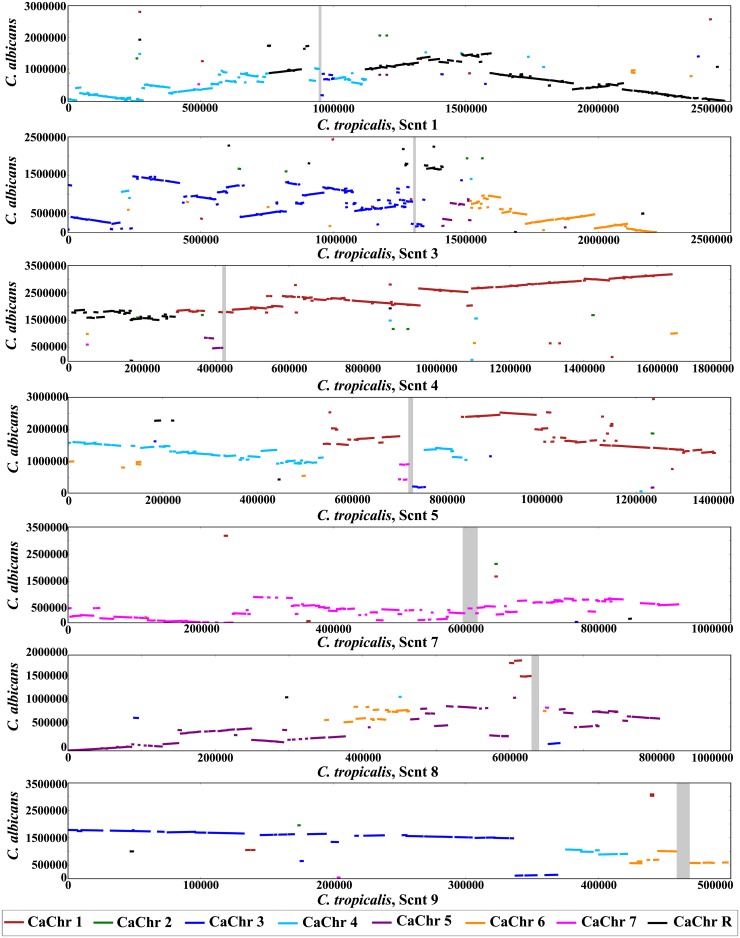
Inter-chromosomal rearrangements at the centromeres of *Candida* species. Orthologous genes are plotted on the *x*-axis as per *C*. *tropicalis* candidates (start to end on that supercontig), and on the *y*-axis as per *C*. *albicans* coordinates for the respective chromosome, and colour-coded according to the *C*. *albicans* chromosome. The vertical grey bar indicates the position of the centromere on the *C*. *tropicalis* supercontig. Continuous segments of lines indicate rows of syntenous genes.

Rapid divergence in the centromere sequence and structure is often associated with karyotypic changes [53, 54], a hallmark of speciation [55–58]. Previously, we demonstrated rapidly changing DNA sequence at the centromeres of orthologous chromosomes in *C*. *albicans* and *C*. *dubliniensis* without any significant changes in synteny across chromosomes [16]. Here we performed a synteny dot plot analysis between *C*. *albicans* and *C*. *tropicalis* genomes. This analysis revealed massive chromosomal rearrangements involving several syntenic breaks happened between these two species. Unusually, it appears that intra-chromosomal transpositions and inversions are far more common than inter-chromosome recombination (Fig 5). Strikingly, inter-chromosome recombination, though uncommon, tends to occur more often near the centromeres (Fig 5 and S6A Fig). For example, in CtScnt3, the large number of genes to the left of the centromere, and a few on the right, map to CaChr3. But immediately after the centromere, there are some segments on CtScnt3 that are in synteny with CaChrR and CaChr5, and then the remainders of CtScnt3 are largely from CaChr6 (Fig 5). Similar patterns of rearrangement can be seen in most other supercontigs also except CtScnt 7. This pattern of rearrangement was plausible probably due to recombination at highly identical sequences of inverted repeats. A similar phenomenon of centromeric repeat-mediated rearrangement and subsequent gain of a chromosome has been observed in two laboratory strains of *S*. *pombe* [59]. Incidentally, there is a change in chromosome number from eight pairs in *C*. *albicans* and *C*. *dubliniensis* to seven pairs in *C*. *tropicalis* indicating a possible structural rearrangement involving centromere which might have given rise to a centromere gain or loss (S6B Fig). However, CtScnt7 comes almost entirely from CaChr7, but has been heavily rearranged (Fig 5). The unusual preponderance of intra-chromosomal transposition and reversal compared to the smaller numbers of inter-chromosomal translocation may merit further study.

In addition, a putative retrotransposon present at the centromere in *C*. *tropicalis* is found to be conserved at *CEN7* in *C*. *dubliniensis* [16] (S5B Fig). A similar retrotransposon was also found to be present within 50 kb region of *CEN7* in *C*. *albicans* (S6A Fig). This putative transposon is a member of the Ty3/Gypsy family but does not present at putative centromeres in any other *Candida* species. These results, together with the conservation in the CENP-A chromatin length, indicate that the centromere position of these related species was shared by a common ancestor and may have undergone chromosomal rearrangement involving the centromeres of more than one chromosome during evolution.

### *C*. *tropicalis* and *S*. *pombe* share common centromere properties

**Fig 6:**
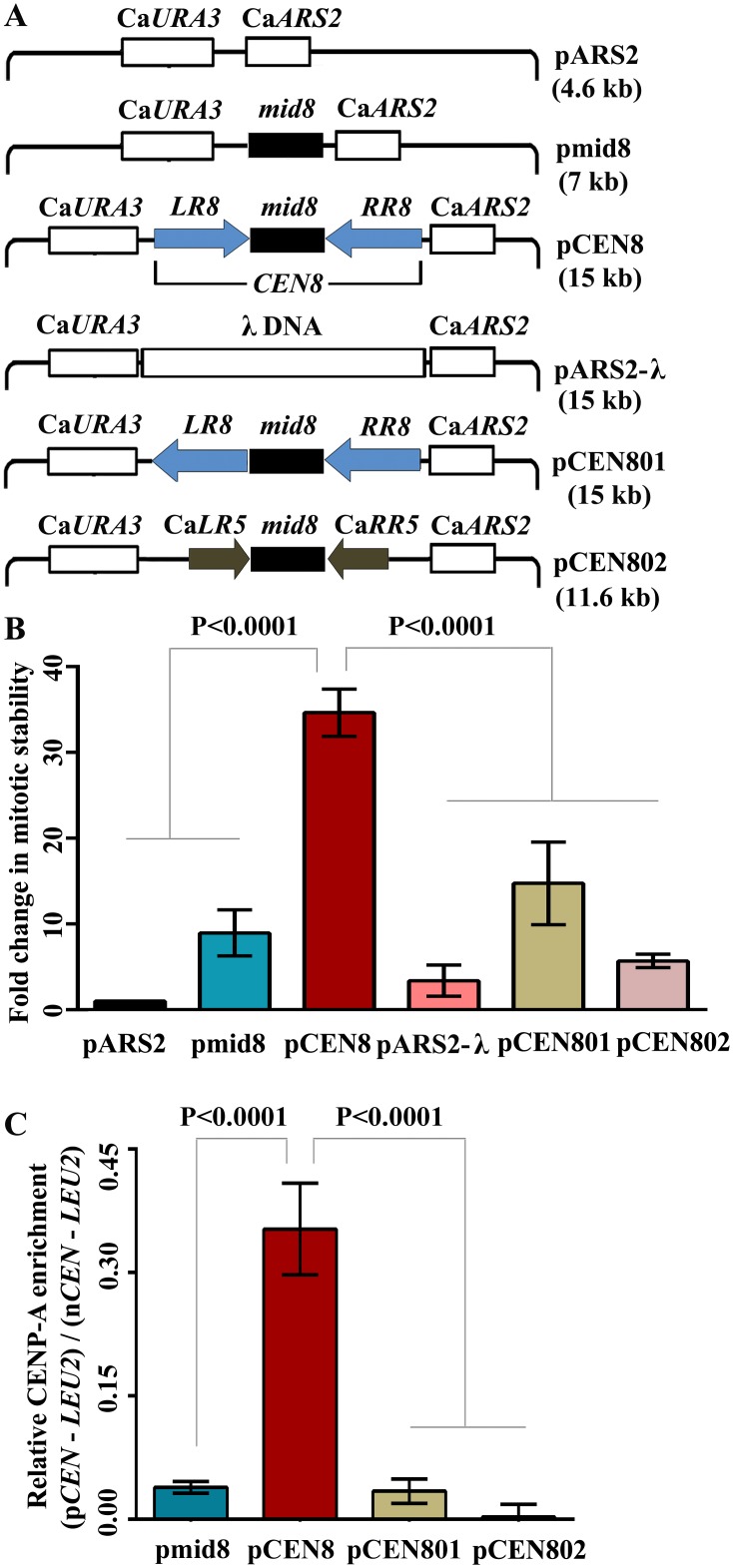
Pericentric inverted repeats provide a structural signature in *de novo* CENP-A recruitment in *C*. *tropicalis*. (A) Schematic of plasmids used in this study. The replicative plasmid pARS2 harbors Ca*ARS2* and Ca*URA3* sequences. pmid8 has only the *mid* core region of *CEN8*, pCEN8 carries the full length centromere (*CEN8*), and pARS2-λ harbors a ~10 kb lambda DNA. pCEN801 carries *LR8* in a direct orientation with respect to *RR8*. On the other hand, pCEN802 harbors Ca*LR5* and Ca*RR5* of chromosome 5 of *C*. *albicans*. The size of each of these plasmids is also mentioned. (B) The relative mitotic stability of various plasmids in *C*. *tropicalis*. The mitotic stability of each of the plasmids is normalized to that of the average mitotic stability of the replicative plasmid (pARS2). The mitotic stability for each class of plasmids was calculated for five independent transformants (n = 5). One way ANOVA and Bonferroni post tests were performed to determine statistical significance. Errors bars represent standard error mean (SEM). (C) CENP-A-ChIP assays were performed in the *C*. *tropicalis* strain CtKS102 (*CSE4/CSE4-TAP*) transformed with pmid8, pCEN8, pCEN801 and pCEN802. Immunoprecipitated (IP) DNA fractions were analyzed by qPCR with primer-pairs (see S4 Table) specific to each cloned insert to determine the extent of *de novo* CENP-A recruitment on the centromere DNA sequence on the plasmid exclusively. The enrichment of CENP-A on these exogenously introduced centromere sequences are represented as a percentage of the total chromatin input with standard error mean (SEM) and validated with three independent biological replicates (n = 3). The relative enrichment was calculated using the formula: (p*CEN*-*LEU2*)/ (n*CEN*-*LEU2*), where n*CEN* and p*CEN* indicate the percent input values of CENP-A enrichment at the native centromere (Scnt 8) and on the plasmid centromere sequence respectively. *LEU2* is used as a non-centromeric negative control. Similarly, one way ANOVA and Bonferroni post tests were performed to determine statistical significance.

The structural features of *C*. *tropicalis* centromeres strikingly resemble those of the distantly related fission yeast *S*. *pombe*. To understand the function of the underlying centromere DNA sequences in *C*. *tropicalis*, we engineered plasmids carrying either the full length centromere (pCEN8) or a part of it (pmid8) on a replicative plasmid pARS2 (Fig 6A). The replicative plasmid pARS2 harbors Ca*ARS2* [60], which functions as an autonomously replicating sequence (*ARS*) on a circular plasmid in *C*. *tropicalis* (S7 Fig). While the pmid8 plasmid conferred 10 to 13-fold increased mitotic stability as compared to pARS2, inclusion of the full length centromere sequence harboring inverted repeats in the pARS2 plasmid (pCEN8) resulted in a 37 to 42-fold higher mitotic stability after 10 generations of nonselective growth (Fig 6B). A size-dependent stabilization of circular replicative plasmids has been reported previously in *S*. *cerevisiae* [61]. To rule out this possibility, we cloned a 10 kb of hererologous DNA sequence from bacteriophage λ (pARS2-λ) and measured the mitotic stability of the same. This plasmid, which is of similar length (15 kb) to that of pCEN8, did not show an increase in the mitotic stability as observed in pCEN8 (Fig 6B). In addition, pCEN8 is 3 to 4-fold more stable mitotically than pmid8 carrying only the *mid* core sequence (Fig 6B). These results suggest that the inverted repeats flanking the *mid* core can significantly improve the mitotic stability of an otherwise unstable replicative plasmid in *C*. *tropicalis*. Because CENP-A is known to bind to only functional centromeres, functionality of a centromere sequence cloned into the replicative plasmid was further assayed by the extent of CENP-A enrichment on these exogenously introduced plasmid DNA constructs. It should be noted that a unique *Sal*I restriction site was introduced at the edge of the *mid* region of the plasmid-borne to differentiate it from the endogenous chromosomal ones (see S8 Fig). CENP-A ChIP-qPCR analysis with plasmid specific primer-pair revealed that CENP-A is enriched at the *mid* core region on only the full length centromere DNA in pCEN8 (Fig 6C) suggesting that the inverted repeats (*LR* and *RR*) flanking the *mid* core are important for *de novo* CENP-A deposition. Thus, we conclude that the CENP-A recruitment process has been significantly rewired in closely related *Candida* species. Centromere function was shown to be dependent on the presence of inverted pericentric repeats in *S*. *pombe* as well [62]. On the other hand, *Candida* species and *S*. *pombe* shared a common ancestor more than 330 mya [63]. Thus, we demonstrate an extraordinary example of evolution of inverted repeat containing ‘fission yeast-like’ centromeres that appeared independently at least once in the *Candida* clade.

### The inverted repeats and the underlying DNA sequence provide a structural determinant for centromere function in *C*. *tropicalis*

In order to find out the role of pericentric inverted repeats and the DNA sequence associated with them for centromere function in *C*. *tropicalis*, we have constructed two different engineered plasmids namely pCEN801 and pCEN802 (Fig 6A). The pCEN801 plasmid harbors the left repeat of *CEN8* (Ct*LR8*) cloned in a direct orientation with respect to the right repeat of the same centromere (Ct*RR8*). Thus, the only difference between pCEN8 (inverted orientation) and pCEN801 (direct orientation) is the orientation of pericentric repeats with respect to each other. However, pCEN801 is found to be significantly less stable mitotically as compared to the pCEN8 (Fig 6B). Moreover, CENP-A ChIP-qPCR analysis revealed that CENP-A does not bind to this engineered pCEN801 plasmid (Fig 6C). These suggest that the inverted orientation of pericentric repeats is an important structural feature for centromere function in *C*. *tropicalis*.

To understand the function of the underlying DNA sequence of the inverted repeats, we cloned the inverted repeats of *CEN5* (Ca*IR5*) from *C*. *albicans* into the pmid8 plasmid (pCEN802). However, the mitotic stability of pCEN802 is found to be 4 to 6-fold lower in *C*. *tropicalis* as compared to pCEN8, which harbors pericentric inverted repeats (*IR8*) of *C*. *tropicalis* (Fig 6B). This observation has been further verified by CENP-A ChIP-qPCR analysis (Fig 6C) and confirms that the sequence of the inverted repeats *per se* is also crucial for centromere function in this species. Thus, we conclude that the DNA sequence of the repeats as well the arrangement of the repeats in an inverted fashion is both important for centromere function in *C*. *tropicalis*.

### The centromeres of *C*. *tropicalis* provide evidence of appearance of pericentric inverted repeats in the Saccharomycotina

**Fig 7:**
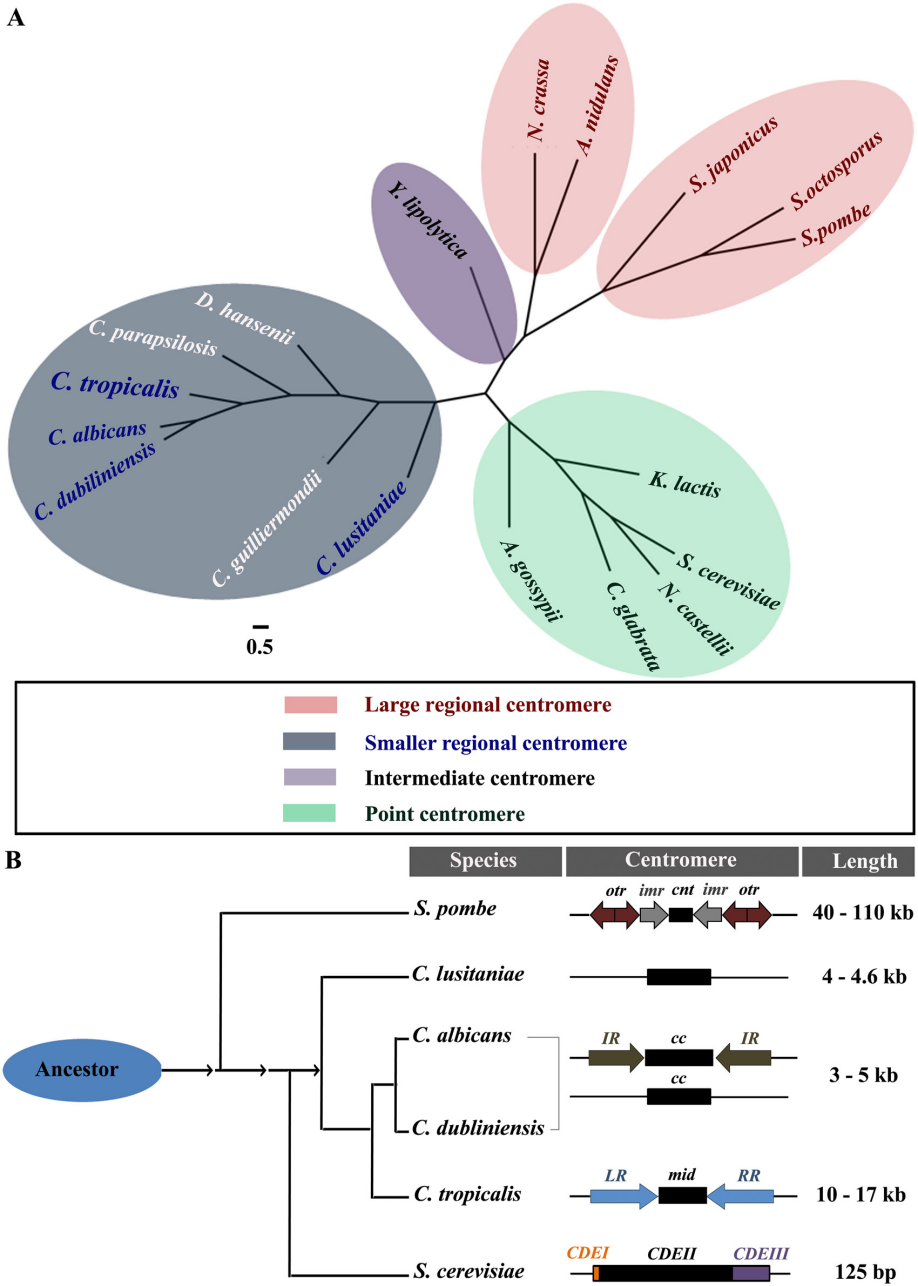
Evolution of centromere organization in ascomycetous fungi. (A) Phylogeny of ascomycetous fungi showing the diverse nature of centromere structure. An unrooted phylogenetic tree was constructed using 573 uniformly evolving orthologous gene families (see methods). The phylogenetic relationship of the species with different types of centromeres is illustrated with colored shadows. The species names shown in white letters designate those with uncharacterized centromeres. The centromeres are mostly point or regional in nature in Ascomycota. However, the centromere of *Y*. *lipolytica* is an example of an unconventional intermediate centromere, which shares properties of both point and regional centromere. The centromeres of *Y*. *lipolytica* are small (<200 bp in size) and mutations in the partial palindrome lead to centromere dysfunction [96]. These are the characteristics of a point centromere. On the other hand, *YlCEN*s lack the conserved DNA elements (*CDE*s). *Y*. *lipolytica* also does not code for the point centromere specific protein complex (the CBF3 complex) [4]. On the other hand, *Y*. *lipolytica* harbors Sim4 and Fta1 proteins, which are kinetochore proteins associated with regional centromere only. Moreover, it should also be noted that the recently identified centromeres of *Naumovozyma castellii* represent an unconventional class of point centromere with unique centromere DNA elements [97]. See text for the detailed information about the classification of the centromere. (B) Schematic shows a possible route of evolution of structural components of the centromere in ascomycetous yeasts. The length of the centromeres is also mentioned. However, it should be noted that the size of the centromeres in *C*. *albicans*, *C*. *dubliniensis* and *C*. *lusitaniae* is based on the length of the CENP-A binding domain. The inverted repeats of *S*. *pombe* and *C*. *tropicalis* centromeres aid in CENP-A recruitment *de novo*. It was also evident from the study in *S*. *pombe* that inverted repeats are essential in the establishment of the centromere, but is no longer required for the maintenance of an already established centromere. On the other hand, the centromeres mostly lack pericentric repeats in *C*. *albicans* where the role of DNA elements in *de novo* CENP-A recruitment is unknown. From these lines of evidence, we propose that the pericentric repeats would have been gradually lost in *C*. *albicans* and *C*. *dubliniensis*. It should also be noted that point centromeres might have originated from the regional ones [98].

To elucidate the route of centromere diversification, we reconstructed a phylogenetic tree of 13 species representing all major lineages of Ascomycota (Fig 7A). It demarcates three distinct monophyletic subphyla within the Ascomycota—Taphrinomycotina, Pezizomycotina and Saccharomycotina (Fig 7A). Moreover, this study also supports that Taphrinomycotina and Pezizomycotina are the early radiating branches in Ascomycetes. Thus, it is evident from both the phylogenetic relationship and the centromere structures of *S*. *pombe* and *N*. *crassa* that the invasion of transposons or symmetric repetitive elements shaped centromere structure in Taphrinomycotina and Pezizomycotina during an early era of ascomycete evolution (Fig 7A). In contrast, a dramatic reduction in centromere length with a concurrent absence or loss of centromeric transposons or repeats, is evidenced from the centromeres of *Candida* and *Saccharomyces* species, and, therefore, evolved in Saccharomycotina (Fig 7A). The identification of the centromere in *C*. *tropicalis* in this study is the first report that shows the evolution of repeat-associated centromeres in the clade of Saccharomycotina (Fig 7A).

## Discussion

In this study, we identified and analyzed the centromeres in *C*. *tropicalis*. We demonstrate that each centromere consists of a central non-repetitive *mid* core region, which is bound by evolutionarily conserved proteins from various layers of the kinetochore, and is flanked by inverted repeats. This is the first known saccharomycetous yeast in which all seven native centromeres are repeat-associated. Moreover, the inverted repeats of the same chromosome as well as across different chromosomes of *C*. *tropicalis* are highly similar in sequence. Taking together these centromere properties of *C*. *tropicalis* and those of other saccharomycetous yeasts, it is now evident that centromeres of all types—point centromeres with conserved motifs that are < 400 bp in length (as in S*accharomyces cerevisiae*), shorter non-repetitive regional centromeres with unique CENP-A-rich regions of 3 to 5 kb long (as in *C*. *albicans*, *C*. *dubliniensis* and *C*. *lusitaniae*) as well as repeat-associated regional centromeres of 10 to 11 kb (as in *C*. *tropicalis*)–evolved in Saccharomycotina (Fig 7B). Although centromere structures are known in only a limited number of organisms, the discovery of all major types of centromeres in the saccharomycetes makes it a unique sub-phylum for tracing the path of evolution of monocentric chromosomes.

The CENP-A-bound DNA sequence is the most preferred site of kinetochore assembly in an entire chromosome. In spite of sharing conserved motifs among centromeres, the CENP-A- bound DNA sequences are often variable, even in the genetically defined point centromeres of *S*. *cerevisiae*. Intriguingly, a comparative analysis between *S*. *cerevisiae* and its closest relative *Saccharomyces paradoxus*, identified that the CENP-A-bound CDE-II elements are the fastest evolving region of the genomes [64]. Similarly, in *S*. *pombe* flanking repeat sequences are conserved among the different chromosomes but the CENP-A-rich central core sequences are heterogeneous [65]. The most extreme cases of rapid divergence have been observed in the centromeres of *C*. *albicans* [15], *C*. *dubliniensis* [16], and *C*. *lusitaniae* [17], where CENP-A-rich centromere DNA sequences are all unique and different in each species. In contrast, CENP-A is found to be enriched on highly homogenized arrays in most plants, mouse, and humans (reviewed in [7]). Thus, homogenization of CENP-A-bound *mid* core regions in *C*. *tropicalis*, as observed in this study, provides a unique feature of yeast centromeres that is more reminiscent of metazoan centromeres. It has been proposed that transposable elements are a major source of centromeric satellite repeats, which gradually homogenized over time by an unknown mechanism in a metazoan system (reviewed in [66]). We also observed a similar association of a retrotransposon in one centromere in *C*. *tropicalis*. More recently, it has been shown that the CENP-A-bound central core has a sequence feature enabling *de novo* recruitment of CENP-A molecules in *S*. *pombe* [67]. Thus, it will be intriguing to investigate a feature of CENP-A-enriched *mid* core regions in *C*. *tropicalis* that may facilitate CENP-A recruitment.

Centromeres are known to be species-specific as centromeres of one organism do not function even in a related species [68]. Inter-species crosses, mostly in plants, suggest that functional incompatibility of centromeres is a frequent cause of uniparental genome elimination [69–71]. Recently, it has been reported that perturbation of the length of the CENP-A binding domain to adopt a uniform size is a prerequisite for a successful inter-species hybridization between maize and oat [69]. Thus, the length of the CENP-A-rich region at the centromere may be a key factor for centromere incompatibility in close relatives. In addition, the length of the CENP-A binding domain is found to be uniform in an organism regardless of the chromosome size or the nature of the centromere. Indeed, we observed that the length of the CENP-A binding region (3 to 5 kb) is surprisingly conserved in related *Candida* species, in spite of the dramatic transition in the centromere organization. A uniform length of the CENP-A-bound regions in these related species may thus suggest a possible role in maintaining uniform kinetochore-microtubule interactions. This is further supported by the fact that the Dam1 complex is essential in *C*. *tropicalis*. Essentiality of the Dam1 complex has been previously correlated to a one microtubule-one kinetochore type of interaction as observed in *S*. *cerevisiae* and *C*. *albicans* [44, 46]. Recently, it was proposed that DNA sequence repeats might have evolved to provide a ‘safety buffer’ against drifts in kinetochore position [72]. Interestingly, we found that the binding of kinetochore proteins is restricted to a non-repetitive *mid* core region in all cases in *C*. *tropicalis* and does not spread to the surrounding inverted repeats. CENP-A chromatin is generally repressive (reviewed in [73]) and thus the safety buffer provided by the pericentric inverted repeats perhaps act as a barrier to prevent the drift of kinetochore position and maintain the size of CENP-A binding domain in this organism.

A series of growing lines of evidence suggest that fungal centromeres are rapidly evolving genomic loci (reviewed in [2]). It has been proposed that rapid evolution of centromere DNA may contribute to its functional incompatibility and perhaps aids in speciation [1, 18]. Speciation is, however, a poorly defined and less understood process in asexual organisms [74]. Some *Candida* species with known centromere structures (*C*. *albicans*, *C*. *dubliniensis* and *C*. *tropicalis*) are primarily parasexual and capable of mating but lack a recognized meiotic program. In spite of this, we observed in this study a high degree of divergence in the centromere DNA sequences as well as in the organization of centromere elements in these related *Candida* species. Why does the centromere structure diverge so rapidly in these related organisms? It has been proposed that the loss of centromere function followed by the birth of a centromere in a new position can be viewed as a life cycle of a centromere that operates during evolution (reviewed in [75]). For such an event, massive chromosomal rearrangements including the loss of an existing centromere would have to occur. Coincidentally, a comparative analysis among the relatives of both yeasts [76] and mammals [77] identified frequent breakpoints adjacent to centromeres. These results suggest that centromeres are among the most fragile sites in a genome. We also observed a gross chromosomal rearrangement between *C*. *albicans* and *C*. *tropicalis* specifically at the centromeres. It is also clear that the centromere loss or gain happened in these two organisms during their divergence from a common ancestor. Being both commensal and opportunistic pathogens, *Candida* species show considerable genome plasticity possibly as a means to survive in a hostile host environment. Genome rearrangements including karyotype changes, aneuploidy, and loss of heterozygosity have been frequently observed in clinical isolates of *Candida* species (reviewed in [78]). Thus, it is likely that the evolutionary life cycle of a centromere may have contributed to their rapid divergence in these related pathogenic yeast species.

Evolution is typically thought to proceed to generate diversity [79]. However, independent evolutionary origins of similar biological structures or functions in distantly related taxa challenge this common paradigm [80]. In this study, we observed that structural features of the *C*. *tropicalis* centromeres resemble a shorter version (10 to 11 kb) of the distantly related *S*. *pombe* centromeres (40 to 110 kb) [12, 65]. However, the pericentric inverted repeats observed in *C*. *tropicalis* have no sequence identity to either the pericentric repeats of *S*. *pombe* or the centromere associated inverted repeats of *C*. *albicans* or *C*. *dubliniensis*. A notable difference between the centromeres of *S*. *pombe* and *C*. *tropicalis* is the absence of outer repeats (*otr* in *S*. *pombe*) in *C*. *tropicalis*. The *otr* is the site of small RNA (siRNA) generation and subsequently *otr* recruits other heterochromatin proteins (such as Swi6 and Clr4 in *S*. *pombe*) to make the centromeric region heterochromatic in *S*. *pombe* (reviewed in [81]). Heterochromatin proteins and siRNAs play a vital role in centromere identity in this organism. Unlike *S*. *pombe*, *C*. *tropicalis* genome neither possesses the full RNAi machinery nor several key players required for heterochromatin formation such as an ortholog of Clr4 (H3K9 methyltransferase) [82]. Thus involvement of repeat elements in establishing RNAi-dependent H3K9me heterochromatin formation, as observed in *S*. *pombe*, is unlikely in *C*. *tropicalis*. In conclusion, we demonstrate for the first time the evolution of repeat-associated centromeres in an ascomycetous budding yeast (Fig 7B). The most reasonable explanation for the appearance of the repeat-associated centromere structure is the contribution of repeats to *de novo* CENP-A deposition. CENP-A is a universal marker of functional centromeres and does not localize at inactivated centromeres. Studies on artificial CENP-A recruitment, either by direct tethering of CENP-A or its chaperone HJURP (also known as Scm3 in yeasts) to an ectopic locus [83, 84], suggest that *de novo* CENP-A deposition is in general one of the most significant rate limiting steps to the acquisition of centromere function. The process of CENP-A recruitment is known to be regulated by both genetic and epigenetic means (reviewed in [2, 29]). However, neither the DNA elements nor epigenetic factors are conserved across the kingdom implying an astounding flexibility in centromere specification. In this study, we demonstrate that a dramatic transition in centromere organization has rewired the genetic and epigenetic regulation of CENP-A deposition in related species. Thus, the ways in which the genetic and the epigenetic factors are co-evolving to orchestrate *de novo* CENP-A recruitment on a DNA sequence to establish a functional centromere may determine the shape of the centromere structure in an organism.

## Methods

### Media and transformation procedure

*C*. *tropicalis* strains were grown either in YPDU (1% yeast extract/ 2% peptone/ 2% glucose/ 0.010% uracil), or in complete minimal (CM) media unless stated otherwise. *C*. *tropicalis* cells were transformed by the standard lithium acetate method as stated previously [45]. It is important to note that *C*. *tropicalis* requires uracil and not uridine in the medium to supplement the Ura auxotrophy.

### Identification of CENP-A, CENP-C, *NUF2* and *DAD1* genes in *C*. *tropicalis*

The centromeric histone H3 CENP-A homolog in *C*. *tropicalis* [85], was identified in a BLAST analysis using *C*. *albicans* CENP-A (CaCse4) as the query sequence against the *Candida tropicalis* genome [43]. The BLAST analysis revealed that the proteins with high scores (score >213) were the putative CENP-A homologue, CtCse4 (CTRG_02639.3), and histone H3 proteins (CTRG_04732.3, CTRG_00676.3 and CTRG_05645.3). The CtCse4 (Scnt 3: 1334129–1334845) is a 238-aa-long protein that shows 90% homology with the C-terminal histone fold domain of CaCse4 (S1A Fig). Similarly, CENP-C (Mif2), Nuf2, and Dad1 homologs of *C*. *tropicalis* were identified in a BLAST analysis. The CtMif2 (CTRG_05763.3) is a 523-aa-long protein (Scnt 9: 474053–475624+) with a conserved CENP-C box, which is identical in sequence between the CaMif2 and CtMif2 (S1A Fig). Ct*NUF2* (CTRG_05381.3) and Ct*DAD1* (CTRG_03625.3) encode 492-aa- and 99-aa-long proteins respectively. Both of these proteins show a high degree of sequence conservation in comparison to those of *C*. *albicans* (S1A Fig).

### Identification of Ct*GAL1* promoter (Ct*GAL1* Pr.) in *C*. *tropicalis*

The sequence upstream of the *GAL1* gene in *S*. *cerevisiae*, harboring the upstream activation sequence (UAS), is used as the *GAL1* promoter to regulate the expression of desired genes [86, 87]. However, no such regulatable promoter has been identified previously in *C*. *tropicalis* to control the expression level and study the essentiality of proteins. The *C*. *tropicalis* homolog of *GAL1* was identified as the ORF (CTRG_04617) by BLAST using *S*. *cerevisiae GAL1* as the query sequence. Further, on analyzing the genomic location of this gene, we found that the synteny of *GAL1* and *GAL10* genes was maintained as observed in *S*. *cerevisiae*.

### Strains construction

The primer sequences and all *C*. *tropicalis* strains used in this study are listed in S4 and S5 Tables respectively. The detailed information about the strain construction is available in the S1 Text.

### Fluorescence microscopy

Cells of *C*. *tropicalis* strains expressing GFP tagged kinetochore proteins were grown overnight, harvested, and washed twice with sterile distilled water. Cells were then resuspended into sterile distilled water to obtain the desired density before taking the images with a Delta Vision Microscopy Imaging system. Indirect immunofluorescence was done as described before [45]. Asynchronously grown *C*. *tropicalis* cells were fixed with a 1/10th volume of formaldehyde (37%) for 1 h at room temperature. Antibodies used were diluted as follows: 1:500 for rabbit anti-Cse4 antibodies [45] and 1:30 for rat anti-tubulin antibodies (Abcam, Cat No. ab6161). The dilutions for secondary antibodies used were Alexa flour 568 goat anti-rabbit IgG (Invitrogen, Cat No. A11011) 1:500 and Alexa fluor 488 goat anti-rat IgG (Invitrogen, Cat No. A11006) 1:500. DAPI (4, 6-Diamino-2-phenylindole) (D9542 Sigma) was used to stain the nuclei of the cells. Cells were examined under 100 (multi) magnifications using a confocal laser scanning microscope (LSM 510 META, Carl Zeiss). The digital images were processed with Adobe Photoshop.

### Flow cytometry (FACS) analysis

*C*. *tropicalis* cells were harvested at two different time points and processed as described before [45]. Prior to injection of the sample into the flow cytometer, the cell suspension was sonicated briefly (30% amplitude, 7s pulse). The sonicated samples were diluted to a desired cell density in 1X PBS and injected into the flow cytometer (BD FACSCalibur) for analysis. The output was analyzed using the BD CellQuestPro software.

### DAPI staining

The conditional mutant strains of *C*. *tropicalis* grown in both permissive and non-permissive media were harvested, washed, and resuspended in 300 μl of sterile distilled water. These cells were fixed by adding 700 μl absolute ethanol and incubated at room temperature for 1 h. After fixing, the cells were washed with 1ml of sterile distilled water twice and resuspended in sterile distilled water to obtain desired cell density prior to imaging. To 5 μl cell suspension, 3 μl DAPI (100 ng/ml) was added in the well, mixed gently by pipetting, and the cover slip was then placed. After 5 min of incubation, the cells were imaged using a fluorescence microscope (Olympus BX51) under 100 (multi) magnifications.

### Western blot analysis

*C*. *tropicalis* strains were grown overnight in YPDU and cells were harvested. The harvested cells were washed with lysis buffer (0.2 M Tris, 1 mM EDTA, 0.39 M ammonium sulphate, 4.9 mM magnesium sulphate, 20% glycerol, 0.95% acetic acid, pH 7.8) and resuspended in 0.5 ml of the same buffer. The cells were disrupted using acid-washed glass beads (Sigma, Cat. No. G8772) by vortexing 5 min (1 min vortexing followed by 1 min cooling on ice) at 4°C. *C*. *tropicalis* cell lysates were electrophoresed on a 12% SDS-PAGE gel and blotted onto a nitrocellulose membrane in a semi-dry apparatus (Bio-Rad). The blotted membranes were blocked with 5% skim milk containing 1X PBS (pH 7.4) for 1 h at room temperature and were then incubated with following dilutions of primary antibodies: anti-Cse4 antibodies [45] 1:500; anti-H3 antibodies [Abcam, Cat No. ab1791] 1:2500; for 1 h at room temperature. Next, the membranes were washed three times with PBST (0.1% Tween-20 in 1X PBS) solution. Anti-rabbit HRP conjugated antibodies [Bangalore Genei, Cat No. 105499] in 1:1000 dilutions were added and incubated for 1 h at room temperature followed by three to four washes with the PBST solution. Signals were detected using the chemiluminescence method (SuperSignal West Pico Chemiluminescent substrate, Thermo scientific, Cat No. 34080).

### ChIP assays and antibodies

The ChIP assays were done as described previously [15]. Briefly, each strain was grown until exponential phase (~2×10^7^ cells/ml) and cells were cross-linked with formaldehyde (final concentration 1%). Chromatin was isolated and sonicated to yield an average fragment size of 300–500 bp. Then the DNA was immunoprecipitated with anti-Cse4 antibodies [45] (final concentration is 6 μg/ml) or anti-protein A antibodies (final concentration is 24 μg/ml) or anti-V5 antibodies (Life Technologies, Cat No. R960-25) (final concentration is 0.94μl/ml) and purified. The duration of cross-linking varies—15 min for CENP-A, 20 min for CENP-C, 1 h 45 min for Nuf2 and 3 h 15 min for Dad1. The total, immunoprecipitated (IP) DNA, and beads only material were used to determine the binding of kinetochore proteins in all seven putative centromeres by semi-quantitative PCR. PCR conditions for primers (as listed in S5 Table) were used as follows: 94°C for 2 min, Tm for 30 s (Tm varies with the primers), 72°C for 1 min, for 1 cycle; 94°C for 30 s, Tm for 30 s, 72°C for 1 min for 24 cycles in case of CENP-A and CENP-C; and 27 cycles for Nuf2 and Dad1; 72°C for 10 min.

### ChIP sequencing, Sanger sequencing and analysis

ChIP-seq analyses were conducted as described previously [23]. The detailed procedure of ChIP-seq and analysis are provided in the S1 Text.

### Pulsed field gel electrophoresis

*C*. *tropicalis* strain MYA-3404 was grown until exponential phase (~2×10^7^ cells/ml). Cells were washed with 50 mM EDTA and counted with a hemocytometer. Approximately 6×10^8^ cells were used for the preparation of 1 ml genomic DNA plugs. The plugs were made according to the instruction manual protocol (BioRad, Cat No. 170–3593) with cleancut agarose (0.6%) and the lyticase enzyme provided by the kit. A 0.6% pulsed field certified agarose gel was prepared using 0.5X TBE buffer (0.1 M Tris, 0.09 M boric acid, 0.01 M EDTA, pH 8) and the PFGE was performed on a CHEF-DR II (Bio-Rad) for 72 h (24 h at 4.5 V/cm/106° with an initial and final switch times 200 s; 48 h at 3 V/cm/106° with an initial and final switch time 700 s). The gel was stained with ethidium bromide (EtBr) and analyzed by using the Quantity One software (Bio-Rad).

### Quantitative PCR (qPCR)

To determine the extent of binding of kinetochore proteins on the centromere of Scnt 8, real time PCR (qPCR) was performed. The template used was as follows: 1 μl of 1:100 dilutions for input and 1 μl of 1:5 dilutions for IP. The conditions used in qPCR were as follows: 94°C for 2 min; 94°C for 30 s, Tm for 30 s (Tm varies with the primers), 72°C for 45 s for 30 cycles. The results were plotted on a graph according to the percentage input method using the formula: 100*2^ (adjusted Ct input−adjusted Ct of IP). Here, the adjusted value is the dilution factor (log_2_ of dilution factor) subtracted from the Ct value of diluted input or IP [88]. Similar conditions were used to determine the enrichment of CENP-A proteins on the centromeric plasmids.

### Computational analysis

To determine conservation rates for inverted repeats (*IR*s) within and across centromeres, and *mid* regions across different centromeres, we used Sigma version 2 [89], a program that aims to minimize spurious alignments by using a stringent p-value for all local alignments, and uses a background model with correlations and an evolutionary model to link sequences. The background model and substitution matrix were drawn from *S*. *cerevisiae* and close relatives, and are not expected to vary significantly across Saccharomycetes. The branch lengths were determined dynamically. The conservation rates in Fig 4C were determined from these alignments using custom python scripts. The visual representation of the alignments as shown in S5A, S5C and S5D Fig was created with an in-house program. The dotplot in S5E Fig was created with dotmatcher, from EMBOSS 6.3.1 [90]. Additionally, the inverted repeats and *mid* regions were scanned for tandem repeats using the Tandem Repeats Finder version 4.04 [91]. The parameters used were “filename 2 5 5 80 10 2 2000” (maximum period size 2000). The results are summarized in S3 Table. For the synteny analysis as in Fig 5, orthology information was obtained from the Fungal Orthogroups Repository (http://www.broadinstitute.org/regev/orthogroups) [92]. Genes in each species within 100 kb of each centromere were examined, and orthologous genes were plotted using an in-house program.

### *ARS* plasmid assay

Approximately 1 μg of DNA of both pARS2 and the control parental (pUC19-Ca*URA3*) plasmids were used to transform CtKS04 strain using both the lithium acetate and the spheroplast transformation methods as stated before [93]. After transformation, the cells were plated on the complete media lacking uracil (CM-Ura) and incubated at 30°C for 3 to 5 days before taking photographs. The *ARS* activity of pARS2 was determined as the transformation efficiency (i.e., the number of transformants/ μg of DNA). Each transformation was performed at least 3 times.

### Mitotic stability assay

The mitotic stability assay was performed to determine the loss rate of pARS2, pmid8, pCEN8, pARS2-λ, pCEN801 and pCEN802 in *C*. *tropicalis*. Briefly, the *C*. *tropicalis* strain, CtKS102 transformed with above mentioned plasmids were streaked on CM-Ura plates for single colonies. Single colonies thus obtained were subsequently inoculated in a nonselective media (YPDU) and incubated at 30°C for overnight for at least 10 generations. Next day, equal numbers of cells were simultaneously plated on YPDU and CM-Ura and incubated at 30°C for 2 days. Colonies grown on both plates were counted and the mitotic stability was calculated in percentage as follows: Mitotic stability = (S/NS), where S and NS denote the number of colonies grown on selective and nonselective media respectively.

### Phylogenetic analysis

A phylogenetic tree with estimated geological time was created via a multiple alignment of 573 gene orthologue sets in 13 sequenced species of Ascomycetous fungi (as shown in Fig 7A)–namely, *C*. *tropicalis*, *S*. *cerevisiae*, *C*. *glabrata*, *K*. *lactis*, *A*. *gossypii*, *N*. *casetelli*, *C*. *dubliniensis*, *C*. *albicans*, *C*. *lusitaniae*, *D*. *hansenii*, *C*. *guilliermondii*, *Y*. *lipolytica*, *N*. *crassa*, *A*. *nidulans*, *S*. *japonicus*, *S*. *octosporus*, *S*. *pombe*. The orthologous genes were identified using the Fungal Orthogroups Repository (http://www.broadinstitute.org/regev/orthogroups/) [92], except in the case of *C*. *dubliniensis* for which orthologues to *C*. *albicans* were used as annotated in the gene sequences from the *Candida* Genome Database (http://www.candidagenome.org/). Only genes for which there were unique reciprocal orthologues between *S*. *cerevisiae* and each of the 13 other species, and which lacked introns (or from which we could easily remove introns) were considered. To remove bias from outliers, the orthologous genes in all species were further sub-selected for genes that evolve uniformly. For this, the average rates of synonymous (ds) and non-synonymous (dn) substitution were calculated separately from codon-level alignments. Only genes whose ds and dn both fell within 1.5 standard deviations of the mean for the full set were considered. This yielded a list of 573 genes. These coding DNA sequences were aligned at the codon level with FSA [94] (command line option “—translated”), concatenated with gaps removed, and a tree was generated with codonphyml [95] (command line “-d codon -q”). Since the quantity of sequence was very large (nearly 10 Mbp, or over 0.5 Mbp per species) bootstrapping was not done.

## Supporting Information

S1 Text Supplemental text discussion and detailed methods. (DOCX)

S1 Fig Kinetochore proteins in *C*. *tropicalis*. (A) A pair-wise alignment of each of the four putative kinetochore proteins in *C*. *albicans* and *C*. *tropicalis*. The domain architecture of CENP-A and CENP-C is shown below the sequence alignment. (B) A pair-wise alignment of first 18 amino acids of CaCENP-A and CtCENP-A as shown in above panel revealed a high level of amino acid sequence conservation. Anti-Cse4 antibodies were raised against the first 18 amino acids of CaCENP-A. Western blot analysis with anti-Cse4 and anti-histone H3 antibodies was performed to detect the specificity of anti-Cse4 antibodies. (C) CENP-A is localized at the kinetochores in *C*. *tropicalis*. Fixed cells of *C*. *tropicalis* strain MYA-3404 were stained with DAPI, anti-Cse4, and anti-tubulin antibodies. The intense single red dot-like CENP-A signals were observed in DAPI-stained (blue) nuclei at G1 unbudded cells and segregate to become two dots during mitosis. Corresponding spindle structures (green) are shown by co-immunostaining with anti-tubulin antibodies. Scale bar, 5 μm.
(TIF)

S2 Fig Depletion of kinetochore proteins leads to defective nuclear segregation in *C*. *tropicalis*. Cells of respective conditional mutant strains grown in the repressive condition (glucose) were stained with DAPI and images were taken by fluorescence microscopy. Arrows indicate an unsegregated mass of nucleus at the mother bud neck, which is a typical feature of G2/M arrest in yeasts. The corresponding panels show the DIC images. Scale bar, 5 μm.
(TIF)

S3 Fig Genome-wide CENP-A and CENP-C ChIP-seq analyses in *C*. *tropicalis*. (A) Plots of CENP-A and CENP-C ChIP-seq reads along individual supercontigs of *C*. *tropicalis*. The *x*-axis and *y*-axis represent the coordinates of the chromosomal regions and the distribution of sequence reads of the specific supercontig respectively as described before. However, it should be noted that ChIP-seq analysis using standardized protocols detected reads on all supercontigs except Supercontig 19 (Scnt 19) (A supercontig will be referred as ‘Scnt’ and number will indicate the supercontig number). (B) The resequenced portion of Scnt 1 shows regions of high sequence similarity (stretches of a few hundred base pair (bp) with no or very few mismatches) with the original Scnt 1, Scnt 3 and Scnt 19. The similar regions are marked as (i), (ii), (iii) and (iv). This suggested that these two supercontigs share long stretches of nearly identical sequence. Given the assumption of the ChIP-seq analysis algorithm that only allows uniquely aligning reads (see methods), this high degree of identity would cause problems both in the original assembly and in uniquely aligning our ChIP-seq reads. Thus, we have carried out ChIP-seq analysis against a reference that consisted of all supercontigs except Scnt 19.
(TIF)

S4 Fig Centromeric regions belong to different chromosomes in *C*. *tropicalis*. Chromosomes of *C*. *tropicalis* were resolved on CHEF gels and stained with ethidium bromide (EtBr) along with *C*. *albicans* chromosomes used as size markers (left-most lane). The gels were blotted and probed with unique sequences of the corresponding supercontigs that carry a CENP-A-rich region (right lanes). Southern hybridization shows that each CENP-A-rich centromeric region belongs to a unique chromosomal band in *C*. *tropicalis*. All Southern blot images were derived by reprobing the same membrane.
(TIF)

S5 Fig The centromere of *C*. *tropicalis* comprises of a homogenized *mid* core flanked by inverted repeats. (A) Homologous segments in the middle regions (*mid*s) of the seven centromeres are shown. In addition, the extended length of *mid7* is due to presence of two annotated retrotransposons in *C*. *tropicalis*. (B) The centromeric location of one of these retrotransposons (CTRG_05088.3) and its homolog (Cd36_71790) is conserved between *C*. *tropicalis* (Ct*CEN7*) and *C*. *dubliniensis* (Cd*CEN7*). (C) Homologous segments in the inverted repeat arms of the seven centromeres. The red bars indicate the arms of the inverted repeats, and are drawn to scale. The coloured bands crossing the arms indicate homologous segments. However, when they pass under an arm, it indicates no homology on that arm. '*LR*' or '*RR*' represents the left or right repeats; supercontig numbers are shown on the right. In (A) and (C) asterisk (*) represents a reverse-complementary sequence. (D) Homologous segments in pair-wise alignments of the 7 pairs of inverted repeat arms at the centromeres, demonstrating that the conservation between inverted repeats of the same centromere is significantly higher than across centromeres. (E) A comparison of the inverted repeats of the centromere in Scnt 5 with a dotplot, showing that regions with tandem repeats (squares along the diagonal) tend to correspond with breaks in the sequence alignment. This pattern of three regions of tandem repeats is seen in most arms.
(TIF)

S6 Fig Orthologous genes present across 100 kb of centromeres in *C*. *albicans* and *C*. *tropicalis*. (A) Orthologous genes or groups of genes on the same strand are joined with blue bands, and on the opposite strand with red bands. Genes in grey colour may have orthologs 100 kb or farther from centromeres in these species. Green regions show the centromeres of both the organisms. (B) Phylogeny of *Candida* species with chromosome number present in each species. Phylogeny shown here is adapted from Fitzpatrick *et al*. [99].
(TIF)

S7 Fig Characterization of pARS2 as a replicative plasmid in *C*. *tropicalis*. A map of pARS2 with the cloned sites of Ca*URA3* and Ca*ARS2* is shown. The plate pictures show the *ARS* function assay of pARS2 as compared to control parent plasmid (pUC19-Ca*URA3*). A table shows the transformation frequency of pARS2 as compared to the control as done either by spheroplasting (ST) or by the lithium acetate method (LT). The transformation experiment was done with three replicates (n = 3) and the mean with standard deviation is indicated in each case. The control plasmid did not yield any transformants.
(TIF)

S8 Fig Plasmids used in this study. Schematics represent locations of plasmid specific primer-pairs for each plasmid used in the mitotic stability assays. The brown color demarcates a unique 6-bp *Sal*I site in a plasmid, which is absent at the native locus. The specificity of the amplicon carrying plasmid-borne *CEN8* was achieved by the addition of a unique engineered *Sal*I site at the 3’ end of each primer. Schematics were not drawn to scale.
(TIF)

S1 Table The length and coordinates of the CENP-A and CENP-C binding as identified by the ChIP-seq analysis within an ORF-free region in *C*. *tropicalis*. (DOCX)

S2 Table The length and coordinates of the inverted repeats (*IR*s) along with *mid* core region of each centromere in *C*. *tropicalis*. (DOCX)

S3 Table Tandem repeats (TR) within the *IR*s arms at the pericentric regions, using the Tandem Repeat Finder version 4.04. (DOCX)

S4 Table Primers used in this study. (DOCX)

S5 Table Strains used in this study. (DOCX)
