# Supplementary material for: Repeat-Associated Fission Yeast-Like Regional Centromeres in the Ascomycetous Budding Yeast Candida tropicalis
Source: PLoS Genet. 2016 Feb 4;12(2):e1005839. doi: 10.1371/journal.pgen.1005839 (PMC4741521; doi:10.1371/journal.pgen.1005839)
Supplement: S1 Text — (DOCX) [file pgen.1005839.s001.docx]

**Supplementary Text**

**Repeat-associated fission yeast-like regional centromeres in an ascomycetous budding yeast *Candida tropicalis***

**Gautam Chatterjee, Sundar Ram Sankaranarayanan, Krishnendu Guin, Yogitha Thattikota, Sreedevi Padmanabhan, Rahul Siddharthan, and Kaustuv Sanyal**

**Strains construction**

**Construction of auxotroph strains.**

**Ct*URA3* deletion:** The cassette for the deletion of *URA3* in *C. tropicalis* was based on the *SAT1*-flipper cassette, pSFS2A [1]. A 592 bp of upstream (primer-pair: KDOCt17 and KDOCt18) and a 714 bp of downstream (primer-pair: KDOCt19-KDOCt20) sequences of Ct*URA3* were PCR amplified. The PCR products were digested by *Sac*I and *Sac*II for the upstream, and *Xho*I and *Kpn*I for the downstream sequence. The digested PCR products were cloned into the respective sites of pSFS2A to generate the Ct*URA3* deletion plasmid pURA-del. The deletion cassette, excised by digestion with *Kpn*I and *Sac*I, was used to transform the sequenced strain of *C. tropicalis*, MYA-3404 [2, 3]. Transformants were selected on the plates containing YPDU media with nourseothricin (100 μg/ml) [Werner Bio-agent] (YPDU+NAT). The desired integrants lacking the one copy of Ct*URA3* were confirmed by PCR analysis. Subsequently, the *SAT1* gene was excised out by growing on yeast peptone media with 2% maltose as the sole carbon source (YPMU media) for the FLP recombinase induction to get the nourseothricin-sensitive strain CtKS01 (*URA3/ura3*). The same deletion cassette was used to delete the remaining copy of Ct*URA3*. The *ura3* auxotrophic transformants were screened by inability of growth on complete media plate lacking uracil (CM-Ura). The nourseothricin-sensitive derivative of this strain CtKS02 has both the Ct*URA3* alleles deleted and was confirmed by Southern blot analysis.

**Ct*HIS1* deletion.** A histidine auxotrophic strain was constructed by transforming CtKS02 (*ura3/ura3*) using the *SAT1*-flipper based *HIS1* deletion plasmid pRB55 [4]. The *HIS1* deletion cassette was released by digestion of pRB55 with *Sac*II and *Apa*I. The cassette was transformed to delete one copy of Ct*HIS1* gene and transformants were confirmed positive by PCR analysis. After recycling the cassette to generate nourseothricin-sensitive strain CtKS03 (*ura3/ura3HIS1/his1*), as described before, the remaining copy of Ct*HIS1* was deleted by using the same cassette. The nourseothricin-sensitive strain CtKS04 (*ura3/ura3 his1/his1*) was confirmed by the inability of its growth on complete media without histidine (CM-His) and subsequently by Southern blot analysis.

**Ct*ARG4* deletion.** An arginine auxotrophic was developed similarly as described before using the *SAT1*-flipper cassette, pRB59 [4]. The *ARG4* deletion cassette was released from pRB59 by digestion with *Sac*II and *Apa*I and transformed into CtKS 04 strain to delete the first copy of Ct*ARG4*. The positive integrant was confirmed by PCR analysis and subsequently recycled *SAT1* to become nourseothricin-sensitive strain, CtKS05 (*ura3/ura3 his1/his1 ARG4/arg4*). The only remaining copy of Ct*ARG4* was deleted by the same cassette. The nourseothricin-sensitive strain CtKS06 (*ura3/ura3 his1/his1 arg4/arg4*) was confirmed by an inability to grow on complete media lacking arginine (CM-Arg) and also by Southern blot analysis.

**Construction of GFP tagged strains.** For intracellular localization of kinetochore proteins in *C. tropicalis*, each protein was GFP tagged at its C-terminus by GFP using a GFP tagging plasmid. First, GFP sequence (Ca*GFP*) along with actin terminator sequence was PCR amplified from pNIM1 [5] using the primers SR67 and SR68. The amplicon was digested by *Spe*I and *Xma*I and cloned into the respective sites in pBluescript KS II (pBS) to generate pGFP. Next, Ca*HIS1* was cloned into the *Eco*RI digested pGFP to generate pGFP-HIS.

**CENP-A-GFP:** A 567 bp fragment of CENP-A ORF (CTRG_02639.3) was PCR amplified (primer-pair: CSE4 ORF F and CSE4 ORF R), digested by *Sac*I and *Spe*I and cloned in pGFP-HIS to obtain pCENP-A. A 499 bp sequence downstream of the ORF was amplified (primer-pair: Cse4 DSF and Cse4 DSR) and cloned into *Cla*I and *Xho*I sites of pCENP-A to generate the final construct pCENP-A-GFP. The plasmid was then digested by *Sac*I and *Kpn*I to release the cassette and used to transform CtKS04 to obtain CtKS100 (*CSE4/CSE4-GFP*).

**CENP-C GFP:**  A 486 bp fragment of the CENP-C ORF (CTRG_05763.3) was amplified (primer-pair: KG113-KG119) and cloned into *Sac*II and *Spe*I sites of pGFP-HIS to generate the plasmid pCENP-C-GFP. The plasmid was linearized by *Pme*I and transformed CtKS06 to get the strain CtKS200 (*MIF2/MIF2-GFP*).

***NUF2*-GFP:** A 695 bp fragment of *NUF2* ORF (CTRG_05381.3) was PCR amplified (primer-pair: KG127 and KG128) and cloned into *Sac*II and *Spe*I sites of pGFP-HIS to obtain the plasmid pNUF2-ORF. A sequence downstream of *NUF2* (664 bp, amplified by primers KG129 and KG1) was cloned into *Apa*I and *Kpn*I sites of pNUF2-ORF to generate the plasmid pNUF2-GFP. The plasmid was digested with *Sac*II and *Kpn*I to release the cassette and then transformed CtKS06 to obtain the strain CtKS300 (*NUF2/NUF2-GFP*).

***DAD1*-GFP:** A 290 bp fragment of *DAD1* ORF (CTRG_03625.3) with a unique site *Xba*I was amplified (primer-pair: SR126 and SR127) and cloned into *Sac*II and *Spe*I sites of pGFP-HIS to obtain the plasmid pDAD1-GFP. The plasmid was linearized by *Xba*I and then transformed CtKS04 to obtain CtKS400 (*DAD1/DAD1-GFP*).

Expression of GFP-tagged protein in each strain was confirmed by fluorescence microscopy using a Delta Vision Imaging System (GE).

**Construction of conditional mutant under the Ct*GAL1* promoter.**

**Deletion cassettes for making heterozygous mutants:** The *SAT1* flipper based deletion cassettes were constructed to delete one copy of the ORFs as follows. To delete the first copy of CENP-A, CENP-C, *NUF2* or *DAD1* in *C. tropicalis*, upstream sequences of each gene were PCR amplified (primers VR39 and VR40 for CENP-A, VR41 and VR42 for CENP-C, KG3 and KG4 for *NUF2* and SR25 and SR26 for *DAD1*). The PCR amplified products were digested by *Sac*I and *Sac*II and cloned into respective sites of pSFS2A to generate pCENP-A-Up, pCENP-C-Up, pNUF2-Up and pDAD1-Up respectively. Similarly, the downstream sequences were PCR amplified (primers VR37 and VR38 for CENP-A, VR43 and VR44 for CENP-C, KG1 and KG2 for *NUF2* and SR27 and SR28 for *DAD1*) and the digested DNA sequences were cloned into pCENP-A-Up, pCENP-C-Up, pNUF2-Up and pDAD1-Up to generate pCENP-A-Del, pCENP-C-Del, pNUF2-Del and pDAD1-Del respectively. Each of these plasmids was digested by *Sac*I and *Kpn*I to release the cassettes. The deletion cassettes for CENP-A and *DAD1* were used to transform CtKS04, while that of *NUF2* was used to transform CtKS06. The transformants were selected on YPDU+NAT (100 μg/ml) plates. The positive strains were confirmed by PCR and subsequently, grown in YPMU media to excise the *SAT1* marker. The nourseothricin sensitive heterozygous transformants for CENP-A, *NUF2* and *DAD1* were named CtKS103 (*CSE4/cse4*), CtKS302 (*NUF2/nuf2*) and CtKS402 (*DAD1/dad1*) respectively.

**Construction of a plasmid with Ct*GAL1* promoter (Ct*GAL1* Pr.):** The entire intergenic region (Scnt 6: 769358-770520) between *GAL1* and *GAL10* genes was PCR amplified (primer-pair: SR145.2 and SR146) and cloned as *Cla*I and *Sal*I fragment in pBS-URA to obtain pGAL-URA.

**Cassettes for conditional expression of genes in pGAL-URA:** The upstream sequence of CENP-A (446 bp, amplified using primers VR39 and VR40) and CENP-C (428 bp, amplified by primers VR41 and VR42) were cloned as *Sac*I and *Sac*II fragments in pGAL-URA to obtain pGAL-CENP-A Up and pGAL-CENP-C Up respectively. The ORF sequences of CENP-A (395 bp, amplified using primers VR50 and VR51) and CENP-C (642 bp, amplified by primers VR52 and VR53) were cloned as *Xho*I and *Kpn*I fragments in the respective sites of pGAL-CENP-A Up and pGAL-CENP-C Up to obtain pGAL1 CENP-A and pGAL1 CENP-C respectively. The construct was released by *Sac*I and *Kpn*I digestion and used to transform CtKS103 (*CSE4/cse4*) and CtKS06 to obtain strains CtKS104 (*GAL1*Pr.*-CSE4/cse4*) and CtKS202 (*MIF2/GAL1*Pr.*-MIF2*). To obtain the conditional mutant strain of CENP-C, the *Sac*I and *Kpn*I digested deletion plasmid pCENP-C Del was then used to transform CtKS202 to delete the remaining copy of CENP-C to get nourseothricin-sensitive CtKS203 (*GAL1*Pr.*-MIF2/mif2*). For *NUF2*, a 455 bp ORF sequence with a V5 tag was amplified (primer-pair: KG84 and KG76) and cloned as a *Sal*I and *Kpn*I fragment into pGAL-URA. The resulting plasmid pGAL1 NUF2 was linearized with *Swa*I and was used to transform CtKS302 (*NUF2/nuf2*) to obtain the conditional mutant strain CtKS303 (*GAL1*Pr.*-V5-NUF2/nuf2*). A 473 bp upstream and a 582 bp sequence from the *DAD1* ORF was PCR amplified (primer SR71 and SR72 for US and SR73 and SR74 for DAD1 ORF) and cloned as *Bam*HI and *Pst*I for upstream, and *Sal*I and *Kpn*I fragments for DAD1 ORF into the pGAL-URA plasmid to obtain pGAL1 DAD1. The plasmid was digested by *Bam*HI and *Kpn*I to release the cassette and was used to transform CtKS402 (*DAD1/dad1*) to obtain the conditional mutant CtKS403 (*GAL1*Pr.*-DAD1/dad1*). All the conditional mutants were confirmed by their inability to grow on non-permissive media (YPD containing 2% glucose as sole carbon source).

**Construction of epitope tagged strains.**

**CENP-C-TAP tagging:** A cassette was constructed to tag CENP-C with TAP (Tandem Affinity Purification) tag at the C-terminus by using the overlap PCR strategy. A 529 bp truncated CENP-C (*MIF2*) gene without the stop codon and 702 bp downstream sequence of CENP-C gene were PCR amplified (primer sets CtCENP-C I F and CtCENP-C IV R for ORF ; JNAT-3UTR F and CtCENP-C IIR for downstream). A 777 bp of *TAP* tag and 1216 bp of *NAT1* (Nourseothricin Acetyl Transferase) sequences were amplified from the plasmid pPK335 [6] and plasmid pMG2120 [7] respectively (primers CtCENP-C III F and TAP-JNAT R for TAP tag ; TAP-JNAT F and JNAT-3UTR R for *NAT1*). A 3.2 kb fragment was amplified by overlap PCR with equimolar concentration of all four fragments amplified earlier. The cassette was used to transform MYA-3404 and the nourseothricin-resistant transformants were selected on YPDU+NAT plates. The resulting strain CtKS201 (*MIF2/MIF2-*TAP) was confirmed by Southern blot analysis.

**Dad1-TAP tagging:** The *DAD1* gene with upstream sequences (556 bp, primers DAD1 FP and DAD1 RP) and sequences downstream of DAD1 (472 bp, primers DAD1 3UTR FP and DAD1 3UTR RP) were amplified. The *TAP* with Ca*URA3* fragment was PCR amplified from the plasmid pPK335 (primer-pair: DAD1TAP FP and DAD1 TAP RP). By overlap PCR using an equimolar mixture of these three fragments, a 2.9 kb DAD1-TAP-URA cassette was then amplified. This PCR product was used to transform CtKS04 and transformants were selected on plates containing CM-URA. The resulting strain CtKS401 (*DAD1*/*DAD1*-TAP) was confirmed by PCR.

**CENP-A-TAP tagging:** To tag CENP-A with TAP, an overlap PCR strategy was employed similarly as stated above. The CENP-A (*CSE4*) gene and its downstream sequence were PCR amplified (primers CSE4 1F and CSE4 2R for 594 bp CSE4 ORF and primers CSE4 3UTR F and CSE4 6R for 548 bp DS sequence). The *TAP* with Ca*URA3* fragment was PCR amplified from pPK335 using primers CSE4 3F and CSE4 TAP-R. Using equimolar mixture of these fragments as template, an overlap PCR was setup to amplify a 3 kb CSE4-TAP-URA-DS cassette. The cassette was transformed in CtKS06 strain and transformants were selected on CM-URA. The positive transformants, CtKS101 was confirmed by both PCR and western blot analysis. A cassette to TAP tag CENP-A using *HIS1* marker was constructed as follows. First, Ca*HIS1* was cloned into the *Eco*RI digested pBS to generate pBS-HIS. Then, CENP-A ORF with tagged TAP epitope was amplified from CtKS101 strain (primers KG121 and KG78). The amplified fragment was digested by *Not*I and *Spe*I and cloned into respective sites of pBS-HIS to generate pCENP-A-TAP-HIS. The plasmid was then linearized by *Bst*BI and transformed in CtKS06 to generate CtKS102 (*CSE4*/*CSE4*-TAP). The positive integrant was confirmed by PCR analysis.

**Construction of pmid8, pCEN8, pCEN801 and pCEN802plasmids.**

**pmid8**: To clone the *mid core* region, the *mid8* was PCR amplified (2557 bp, primers SalICConly FP and BamHICConly RP) and digested by *Sal*I and *Bam*HI. The digested DNA was cloned into *Sal*I and *Bam*HI digested pARS2 plasmid to generate pmid8 plasmid.

**pCEN8**: A 6334 bp region including the *mid core* and right inverted repeats (Ct*RR8*) of *CEN8* was PCR amplified (primer-pair: SalICConly FP and CEN8 RP) using *C. tropicalis* genomic DNA as the template and digested by *Sal*I and *Bam*HI. The digested DNA was cloned into respective sites of pARS2 to generate pmid+RR plasmid. A 4032 bp left repeat of *CEN8* (Ct*LR8*) was subsequently PCR amplified (primer-pair: CEN8 FP and SalI IR only RP) using *C. tropicalis* genomic DNA as the template and digested by *Pst*I and *Sal*I. The digested *LR8* amplicon was cloned into *Pst*I and *Sal*I digested pmid8+RR plasmid to generate pCEN8 plasmid.

**pCEN801:** To clone Ct*LR8* into direct orientation with respect to Ct*RR8*, a 4032 bp Ct*LR8* was PCR amplified (primer-pair: KG235 and KG236) using *C. tropicalis* genomic DNA as the template. The PCR product was digested by *Sal*I and *Pst*I and cloned into respective sites of pmid+RR to generate pCEN801. The orientation of Ct*LR8* in pCEN801 was confirmed by *Nco*I digestion.

**pCEN802:** A 2254 bp left repeat of *CEN5* of *C. albicans* (Ca*LR5*) was PCR amplified (primer-pair: KG229 and KG230) using *C. albicans* genomic DNA as the template and digested by *Pst*I and *Sal*I. The digested product was cloned into respective sites of pmid8 to generate pmid8-CaLR5. Subsequently, a 2317 bp right repeat of Ca*CEN5* (Ca*RR5*) was PCR amplified (primer-pair: KG231 and KG232) using *C. albicans* genomic DNA as the template, digested by *Bam*HI and was cloned into the same site of pmid8-CaLR5 to generate pCEN802.

The structure of each of these plasmids was verified by digesting with at least two different enzymes.

**ChIP sequencing and analysis**

Immunoprecipitated (IP) samples for both CENP-A and CENP-C from ChIP experiments as well as the corresponding whole-cell extracts (input) were quantified using Qubit Fluorometer, and the samples with more than 10 ng dsDNA were used for library preparation. Libraries for multiplex ChIP sequencing were constructed using the NEXTflexTMChIP-seq Sample Preparation Kit protocol outlined in “Preparing Samples for ChIP Sequencing of DNA” (BIOO Scientific# IP-5143-01). Briefly, DNA was end filled to repair frayed ends, and also to phosphorylate the fragments. The end repaired fragments are adenylated with a single nucleotide 'A' overhang (BIOO Scientific# IP-5143-01) and ligate adaptors (NEXT Flex adapters). The fragments with ligated adapters are subjected to pre-size selection PCR for 5 cycles followed by size selection on 2% Low Melting Agarose. The size selected samples are enriched with 13 cycles of PCR. The prepared libraries were quantified using Nanodrop Spectrophotometer and validated for quality by running an aliquot on High Sensitivity Bioanalyzer Chip (Agilent).

The DNA from prepared libraries was denatured and sequenced on Illumina Genome Analyzer IIx by Sequencing by synthesis method to read 72 bases single end. DNA library fragments were diluted, denatured and hybridized to a lawn of oligonucleotides immobilized on the flow cell surface. The hybridized DNA template was amplified using immobilized oligonucleotides as primers. Each hybridized template through the process of isothermal bridge amplification resulted in the formation of clusters comprised of roughly 1000 clonal copies. Sequencing was performed by synthesis (SBS) technology using four fluorescently labeled nucleotides to sequence each cluster on the flow cell surface in parallel. During each sequencing cycle, a single labeled deoxynucleotide triphosphate (dNTP) was added and clusters were imaged. The fluorescent dye and blocker was cleaved off and the next complementary base was added to the nucleic acid chain and imaged. A total of 72 such cycles were performed which corresponds to 72 bases sequenced. Individual bases were called directly from signal intensity measurements during each cycle. These cycles comprised Read 1 of the sequencing run. Once sequencing was completed, the raw data was extracted from the server using the proprietary Illumina pipeline software to obtain FASTQ files. Quality check of raw data was performed using an in-house program SeqQC (SeqQC V2.1 - http://genotypic.co.in/SeqQC.html). Reads were aligned to the reference *Candida tropicalis* genome [3] using Bowtie [8] and following parameters were used - i) v 3: reports end to end hits with mismatch <=3, ii) best: hits guaranteed best stratum, iii) m 1: suppress all alignments if > 1, iv) S: write hits in SAM format, v) p 12: number of alignment threads to launch, vi) t: print wall-clock time taken by search phases. Peak calling was performed by Homer [9] with following parameters - i) style histone: histone protein binding sites, ii) F 4: fold enrichment over input tag count, iii) L 4: fold enrichment over local tag count, iv) C 2: fold enrichment limit of expected unique tag positions, v) P 0.0001: poisson p-value threshold relative to input tag count, vi) LP 0.0001: poisson p-value threshold relative to local tag count, vii) poisson 0.002: Set poisson p-value cutoff, viii) fdr 0.0001: False discovery rate.

**Sanger sequencing**

We observed that there is a long stretch of sequence in *CEN1* (the predicted centromeric region in Scnt 1) that is nearly identical to a sequence in Scnt 19 (reverse-complemented). The pair-wise alignment shows 6 mismatches in a gapless local alignment of 2111 bp. Given that this almost identically-repeated region lies adjacent to a stretch of N's (unsequenced) in Scnt 1 (it is the last piece of contig 8), we decided to resequence that region in Scnt 1 to clarify the sequence and rule out possible assembly errors.

We amplified a 3255 bp region of Scnt 1 (Scnt 1: 949444–952698) by high fidelity Phusion taq polymerase and sequenced these regions by the Sanger method with overlapping primers. This includes the approximately 100 bp unsequenced region in *CEN1*. This resequenced region has even stronger identity with the previously sequenced Scnt 19. A 2338 bp of the re-sequenced region of Scnt 1, which includes the newly sequenced region (previously N's), can be aligned with Scnt 19 with only 3 mismatches and no gaps. After this follows a region that aligns almost perfectly with Scnt 3; and a final piece that matches with the original Scnt 1. This suggests that the previously published scaffolds of Scnt 1, 19 and 3 may not be entirely trustworthy (S3B Fig.).

Similarly, we found that large parts of the centromere in Scnt 23 align perfectly with Scnt 9 until it crosses ~1.5 kb unsequenced region of Scnt 9. Thus, we decided to sequence the unsequenced region of Scnt 9 with supercontig specific primer-pair to amplify from Scnt 9. We amplified 4362 bp region from Scnt 9 (Scnt 9: 458541–462903) by high fidelity Phusion taq polymerase. This amplified DNA was used as template to amplify ~1.5 kb DNA and subsequently cloned in TA vector, pTZ 57R/T according to manufacturer's protocol. Then, plasmid specific M13 primer-pair was used to amplify the cloned insert, which was used for Sanger sequencing. The newly sequenced region agrees with the previously published sequence on both sides of the previously gapped region, suggesting that the assembly of Scnt 9 is correct. However, elsewhere there are long (greater than 100 bp) stretches of identical sequence between these supercontigs. In addition, we amplified unsequenced region of Scnt 7 (Scnt 7: 602901-603109), which was used as template for Sanger sequencing to fill up the gap for further studies.

To investigate the problem of multiple identical long sequences in *C. tropicalis*, we did a genome-wide search for such sequences and found that they are indeed abundant. We found 37 cases of identical sequence over 1000 bp long in *C. tropicalis*, compared to only 9 such examples in *C. albicans* (data not shown). This suggests that correct assembly of *C. tropicalis* would be a challenging task.

**References**

1. Reuss O, Vik A, Kolter R, Morschhauser J. The SAT1 flipper, an optimized tool for gene disruption in *Candida albicans*. Gene. 2004;341:119-27. Epub 2004/10/12. doi: S0378111904003555 [pii]

10.1016/j.gene.2004.06.021. PubMed PMID: 15474295.

2. Joly S, Pujol C, SchrÃppel K, Soll DR. Development of two species-specific fingerprinting probes for broad computer-assisted epidemiological studies of *Candida tropicalis*. J Clinical Microbiol. 1996;34(12):3063-71.

3. Butler G, Rasmussen MD, Lin MF, Santos MAS, Sakthikumar S, Munro CA, et al. Evolution of pathogenicity and sexual reproduction in eight *Candida* genomes. Nature. 2009;459(7247):657-62.

4. Porman AM, Alby K, Hirakawa MP, Bennett RJ. Discovery of a phenotypic switch regulating sexual mating in the opportunistic fungal pathogen *Candida tropicalis*. Proc Natl Acad Sci USA. 2011;108(52):21158-63. Epub 2011/12/14. doi: 1112076109 [pii]

10.1073/pnas.1112076109. PubMed PMID: 22158989.

5. Park YN, Morschhauser J. Tetracycline-inducible gene expression and gene deletion in *Candida albicans*. Eukaryot Cell. 2005;4(8):1328-42. Epub 2005/08/10. doi: 4/8/1328 [pii]

10.1128/EC.4.8.1328-1342.2005. PubMed PMID: 16087738.

6. Corvey C, Koetter P, Beckhaus T, Hack J, Hofmann S, Hampel M, et al. Carbon Source-dependent assembly of the Snf1p kinase complex in Candida albicans. J Biol Chem. 2005;280(27):25323-30. Epub 2005/05/14. doi: M503719200 [pii]

10.1074/jbc.M503719200. PubMed PMID: 15890650.

7. Ketel C, Wang HS, McClellan M, Bouchonville K, Selmecki A, Lahav T, et al. Neocentromeres form efficiently at multiple possible loci in *Candida albicans*. PLoS Genet. 2009;5(3):e1000400. Epub 2009/03/07. doi: 10.1371/journal.pgen.1000400. PubMed PMID: 19266018.

8. Langmead B, Salzberg SL. Fast gapped-read alignment with Bowtie 2. Nat Methods. 2012;9(4):357-9. Epub 2012/03/06. doi: nmeth.1923 [pii]

10.1038/nmeth.1923. PubMed PMID: 22388286.

9. Heinz S, Benner C, Spann N, Bertolino E, Lin YC, Laslo P, et al. Simple combinations of lineage-determining transcription factors prime cis-regulatory elements required for macrophage and B cell identities. Mol Cell. 2010;38(4):576-89. Epub 2010/06/02. doi: S1097-2765(10)00366-7 [pii]

10.1016/j.molcel.2010.05.004. PubMed PMID: 20513432.

10. Fitzpatrick DA, Logue ME, Stajich JE, Butler G. A fungal phylogeny based on 42 complete genomes derived from supertree and combined gene analysis. BMC Evol Biol. 2006;6:99. Epub 2006/11/24. doi: 1471-2148-6-99 [pii]

10.1186/1471-2148-6-99. PubMed PMID: 17121679.
