# Supplementary figures and images for: Repeat-Associated Fission Yeast-Like Regional Centromeres in the Ascomycetous Budding Yeast Candida tropicalis
Source: PLoS Genet. 2016 Feb 4;12(2):e1005839. doi: 10.1371/journal.pgen.1005839 (PMC4741521; doi:10.1371/journal.pgen.1005839)

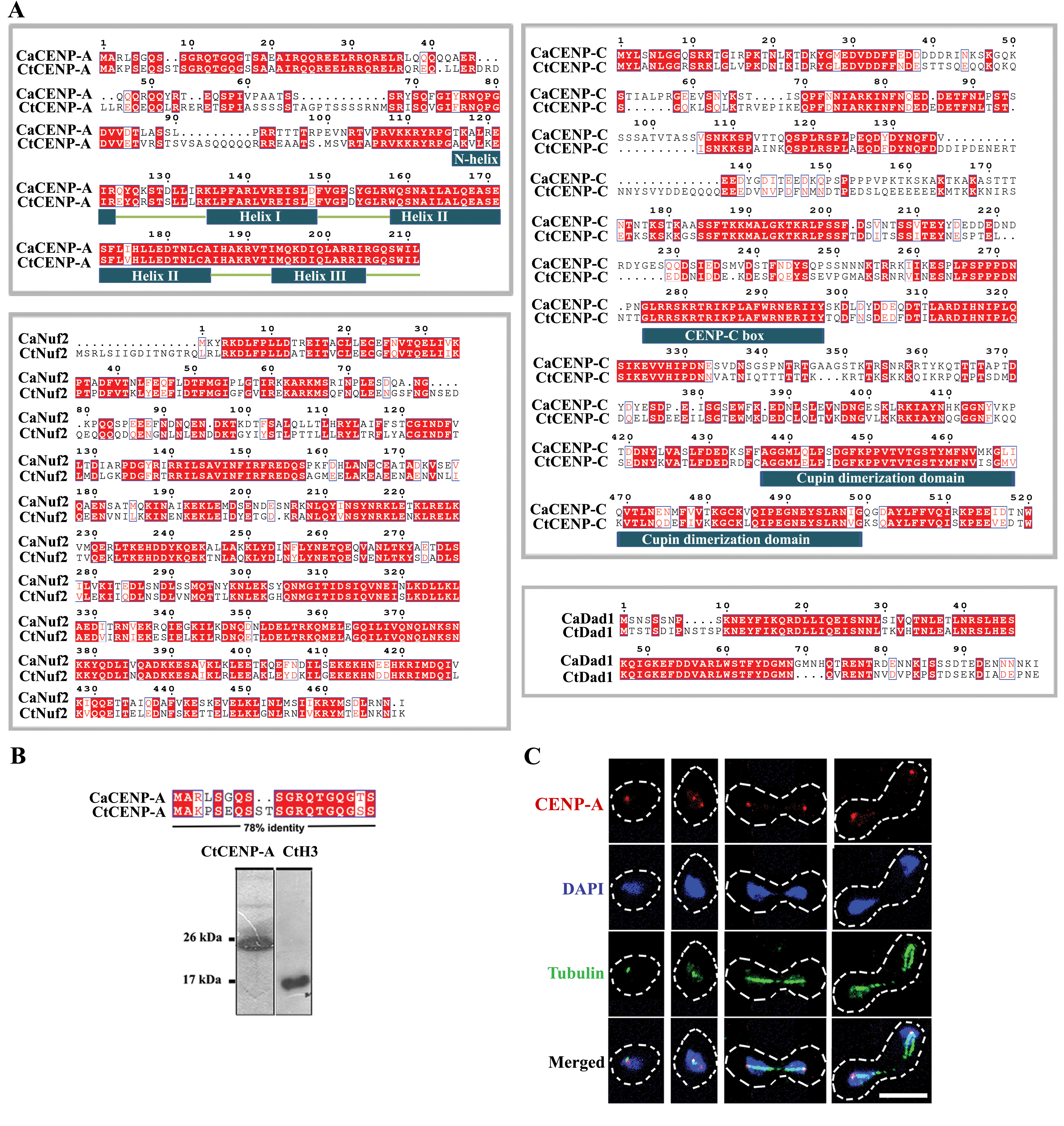

Supplement: S1 Fig — (A) A pair-wise alignment of each of the four putative kinetochore proteins in C. albicans and C. tropicalis. The domain architecture of CENP-A and CENP-C is shown below the sequence alignment. (B) A pair-wise alignment of first 18 amino acids of CaCENP-A and CtCENP-A as shown in above panel revealed a high level of amino acid sequence conservation. Anti-Cse4 antibodies were raised against the first 18 amino acids of CaCENP-A. Western blot analysis with anti-Cse4 and anti-histone H3 antibodies was performed to detect the specificity of anti-Cse4 antibodies. (C) CENP-A is localized at the kinetochores in C. tropicalis. Fixed cells of C. tropicalis strain MYA-3404 were stained with DAPI, anti-Cse4, and anti-tubulin antibodies. The intense single red dot-like CENP-A signals were observed in DAPI-stained (blue) nuclei at G1 unbudded cells and segregate to become two dots during mitosis. Corresponding spindle structures (green) are shown by co-immunostaining with anti-tubulin antibodies. Scale bar, 5 μm. (TIF) [file pgen.1005839.s002.tif]

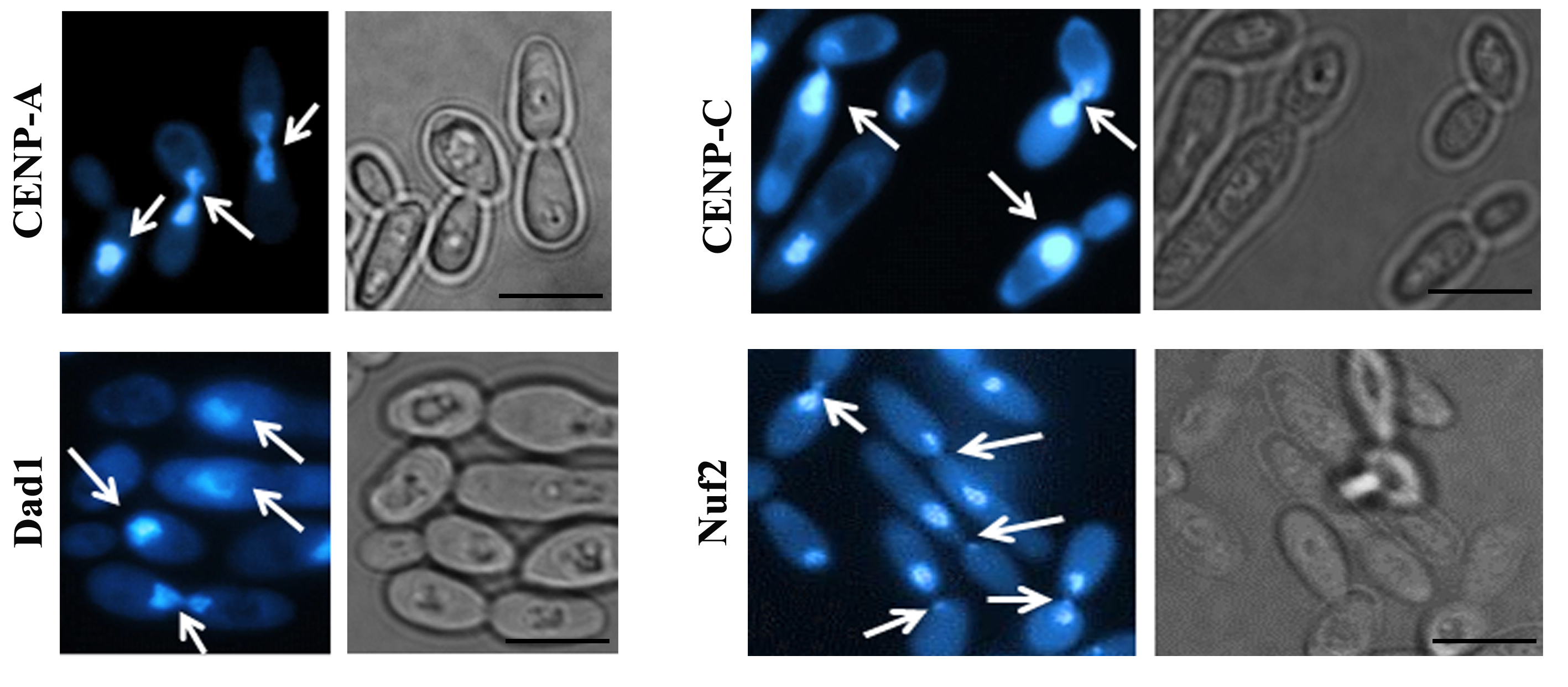

Supplement: S2 Fig — Cells of respective conditional mutant strains grown in the repressive condition (glucose) were stained with DAPI and images were taken by fluorescence microscopy. Arrows indicate an unsegregated mass of nucleus at the mother bud neck, which is a typical feature of G2/M arrest in yeasts. The corresponding panels show the DIC images. Scale bar, 5 μm. (TIF) [file pgen.1005839.s003.tif]

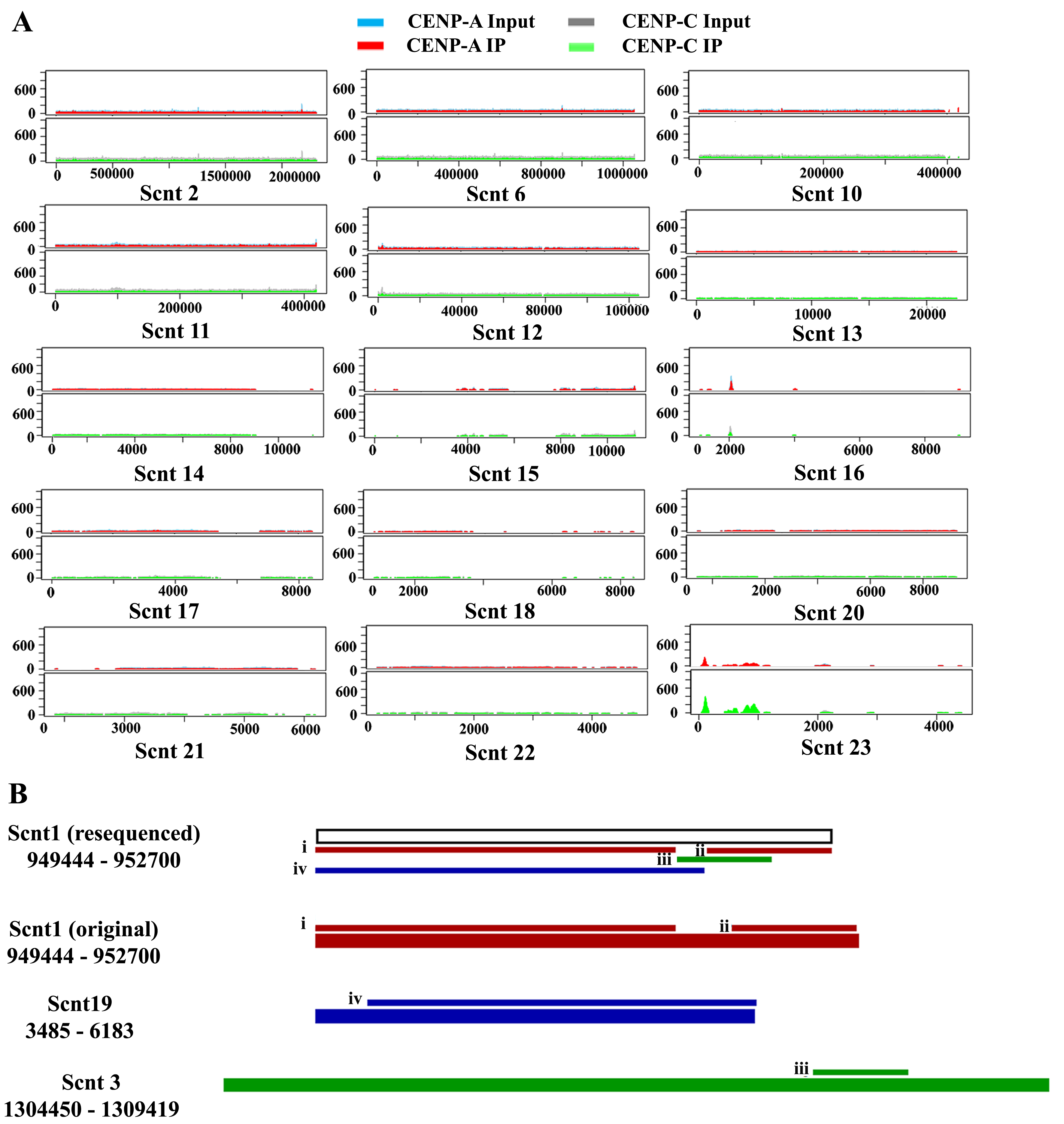

Supplement: S3 Fig — (A) Plots of CENP-A and CENP-C ChIP-seq reads along individual supercontigs of C. tropicalis. The x-axis and y-axis represent the coordinates of the chromosomal regions and the distribution of sequence reads of the specific supercontig respectively as described before. However, it should be noted that ChIP-seq analysis using standardized protocols detected reads on all supercontigs except Supercontig 19 (Scnt 19) (A supercontig will be referred as ‘Scnt’ and number will indicate the supercontig number). (B) The resequenced portion of Scnt 1 shows regions of high sequence similarity (stretches of a few hundred base pair (bp) with no or very few mismatches) with the original Scnt 1, Scnt 3 and Scnt 19. The similar regions are marked as (i), (ii), (iii) and (iv). This suggested that these two supercontigs share long stretches of nearly identical sequence. Given the assumption of the ChIP-seq analysis algorithm that only allows uniquely aligning reads (see methods), this high degree of identity would cause problems both in the original assembly and in uniquely aligning our ChIP-seq reads. Thus, we have carried out ChIP-seq analysis against a reference that consisted of all supercontigs except Scnt 19. (TIF) [file pgen.1005839.s004.tif]

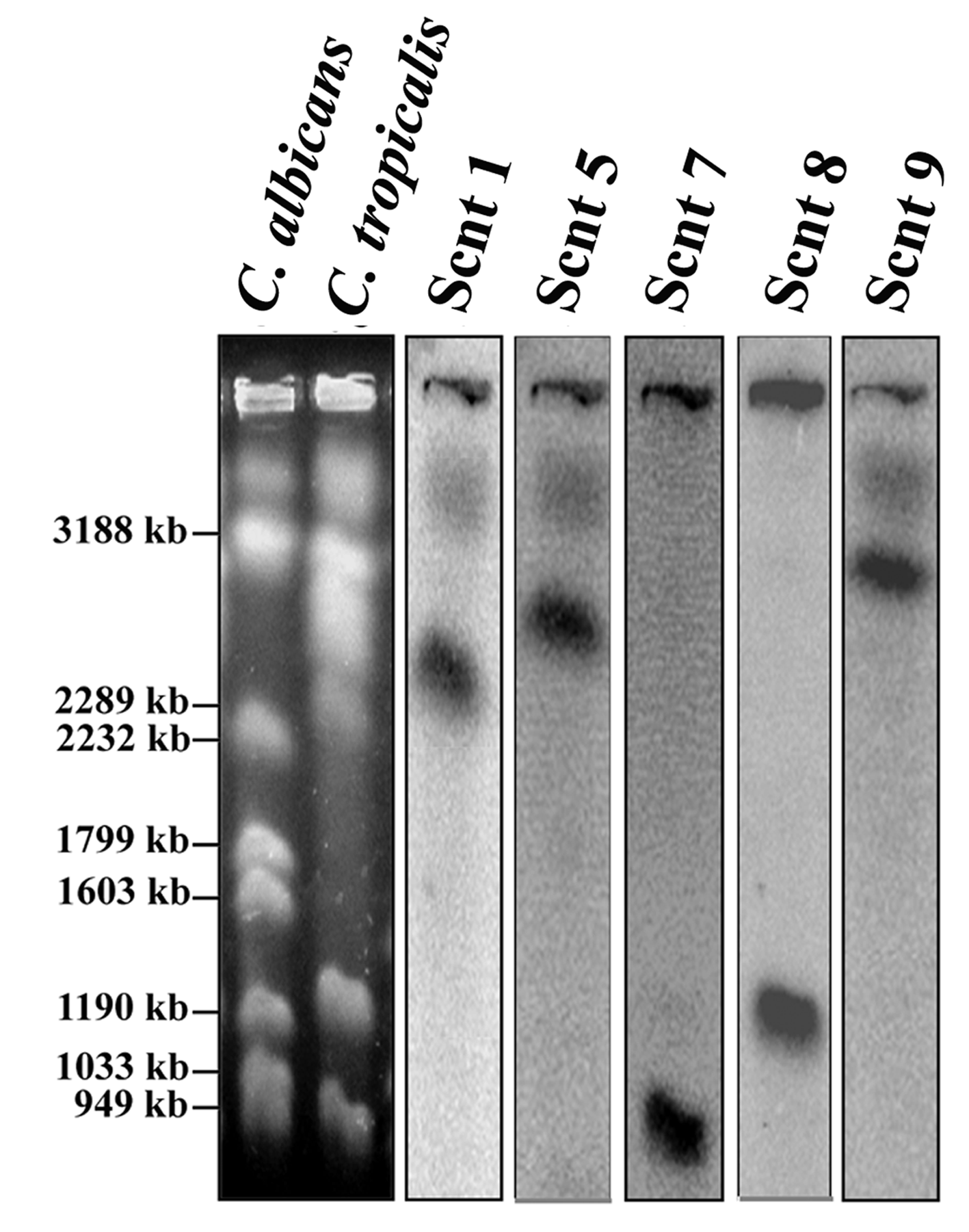

Supplement: S4 Fig — Chromosomes of C. tropicalis were resolved on CHEF gels and stained with ethidium bromide (EtBr) along with C. albicans chromosomes used as size markers (left-most lane). The gels were blotted and probed with unique sequences of the corresponding supercontigs that carry a CENP-A-rich region (right lanes). Southern hybridization shows that each CENP-A-rich centromeric region belongs to a unique chromosomal band in C. tropicalis. All Southern blot images were derived by reprobing the same membrane. (TIF) [file pgen.1005839.s005.tif]

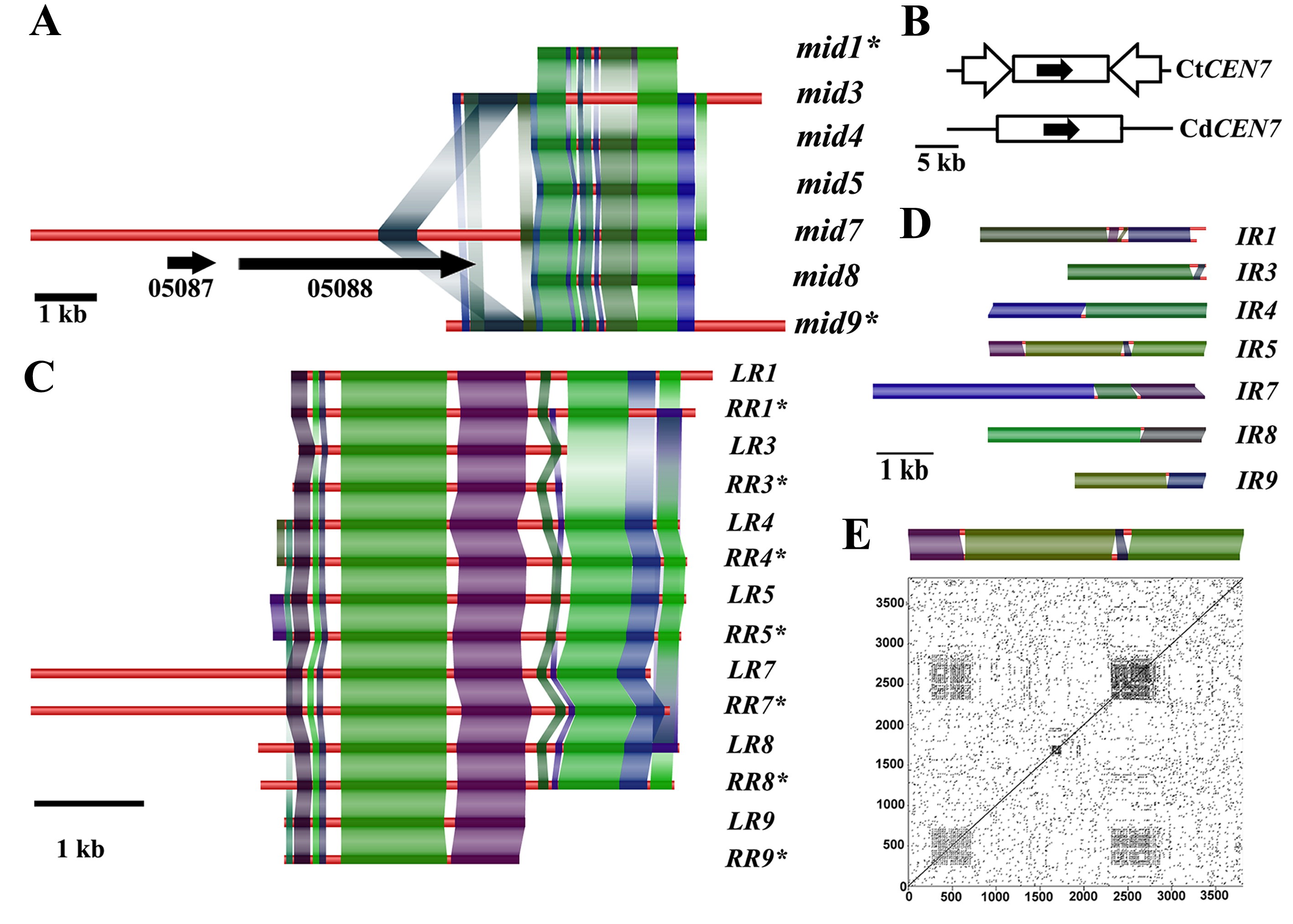

Supplement: S5 Fig — (A) Homologous segments in the middle regions (mids) of the seven centromeres are shown. In addition, the extended length of mid7 is due to presence of two annotated retrotransposons in C. tropicalis. (B) The centromeric location of one of these retrotransposons (CTRG_05088.3) and its homolog (Cd36_71790) is conserved between C. tropicalis (CtCEN7) and C. dubliniensis (CdCEN7). (C) Homologous segments in the inverted repeat arms of the seven centromeres. The red bars indicate the arms of the inverted repeats, and are drawn to scale. The coloured bands crossing the arms indicate homologous segments. However, when they pass under an arm, it indicates no homology on that arm. 'LR' or 'RR' represents the left or right repeats; supercontig numbers are shown on the right. In (A) and (C) asterisk (*) represents a reverse-complementary sequence. (D) Homologous segments in pair-wise alignments of the 7 pairs of inverted repeat arms at the centromeres, demonstrating that the conservation between inverted repeats of the same centromere is significantly higher than across centromeres. (E) A comparison of the inverted repeats of the centromere in Scnt 5 with a dotplot, showing that regions with tandem repeats (squares along the diagonal) tend to correspond with breaks in the sequence alignment. This pattern of three regions of tandem repeats is seen in most arms. (TIF) [file pgen.1005839.s006.tif]

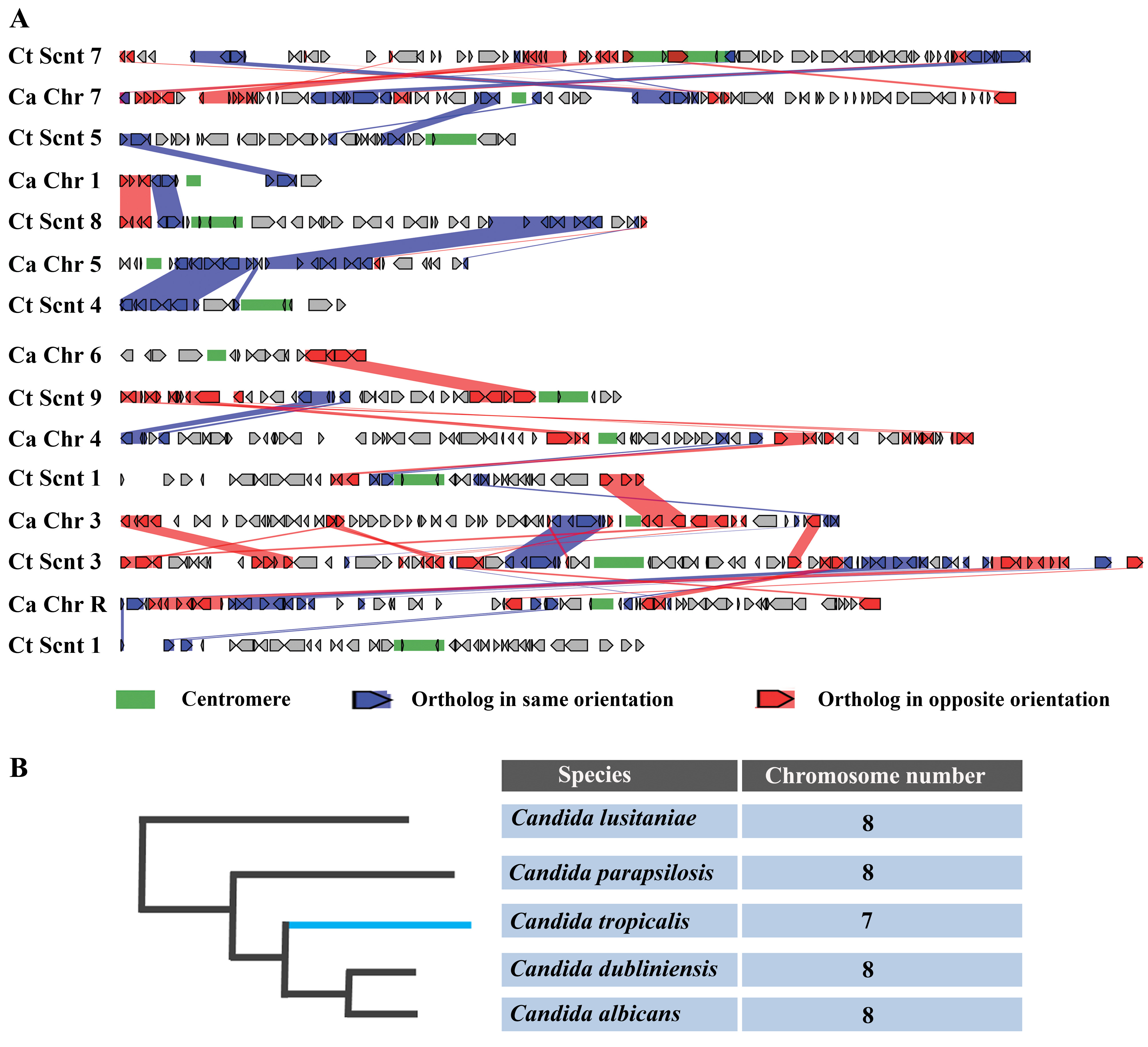

Supplement: S6 Fig — (A) Orthologous genes or groups of genes on the same strand are joined with blue bands, and on the opposite strand with red bands. Genes in grey colour may have orthologs 100 kb or farther from centromeres in these species. Green regions show the centromeres of both the organisms. (B) Phylogeny of Candida species with chromosome number present in each species. Phylogeny shown here is adapted from Fitzpatrick et al. [99]. (TIF) [file pgen.1005839.s007.tif]

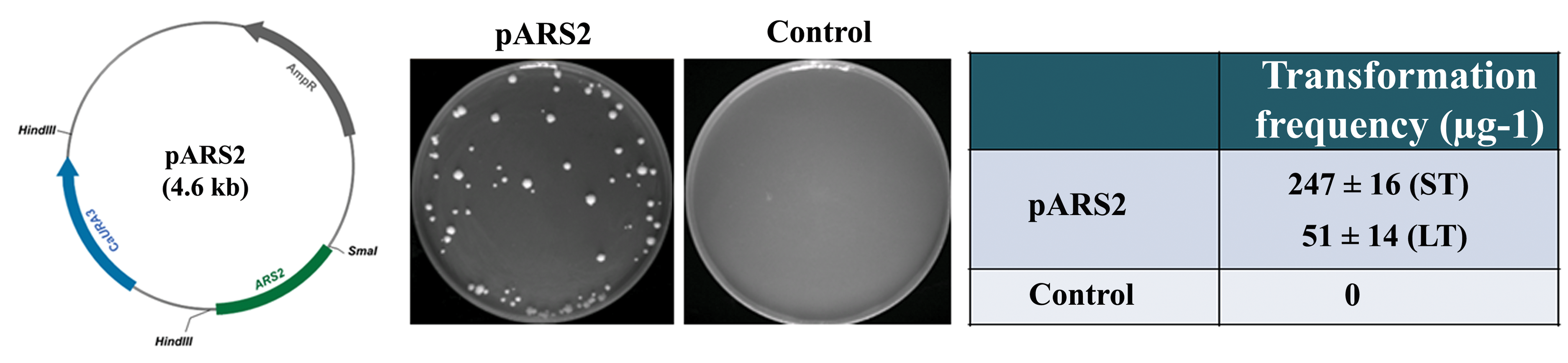

Supplement: S7 Fig — A map of pARS2 with the cloned sites of CaURA3 and CaARS2 is shown. The plate pictures show the ARS function assay of pARS2 as compared to control parent plasmid (pUC19-CaURA3). A table shows the transformation frequency of pARS2 as compared to the control as done either by spheroplasting (ST) or by the lithium acetate method (LT). The transformation experiment was done with three replicates (n = 3) and the mean with standard deviation is indicated in each case. The control plasmid did not yield any transformants. (TIF) [file pgen.1005839.s008.tif]

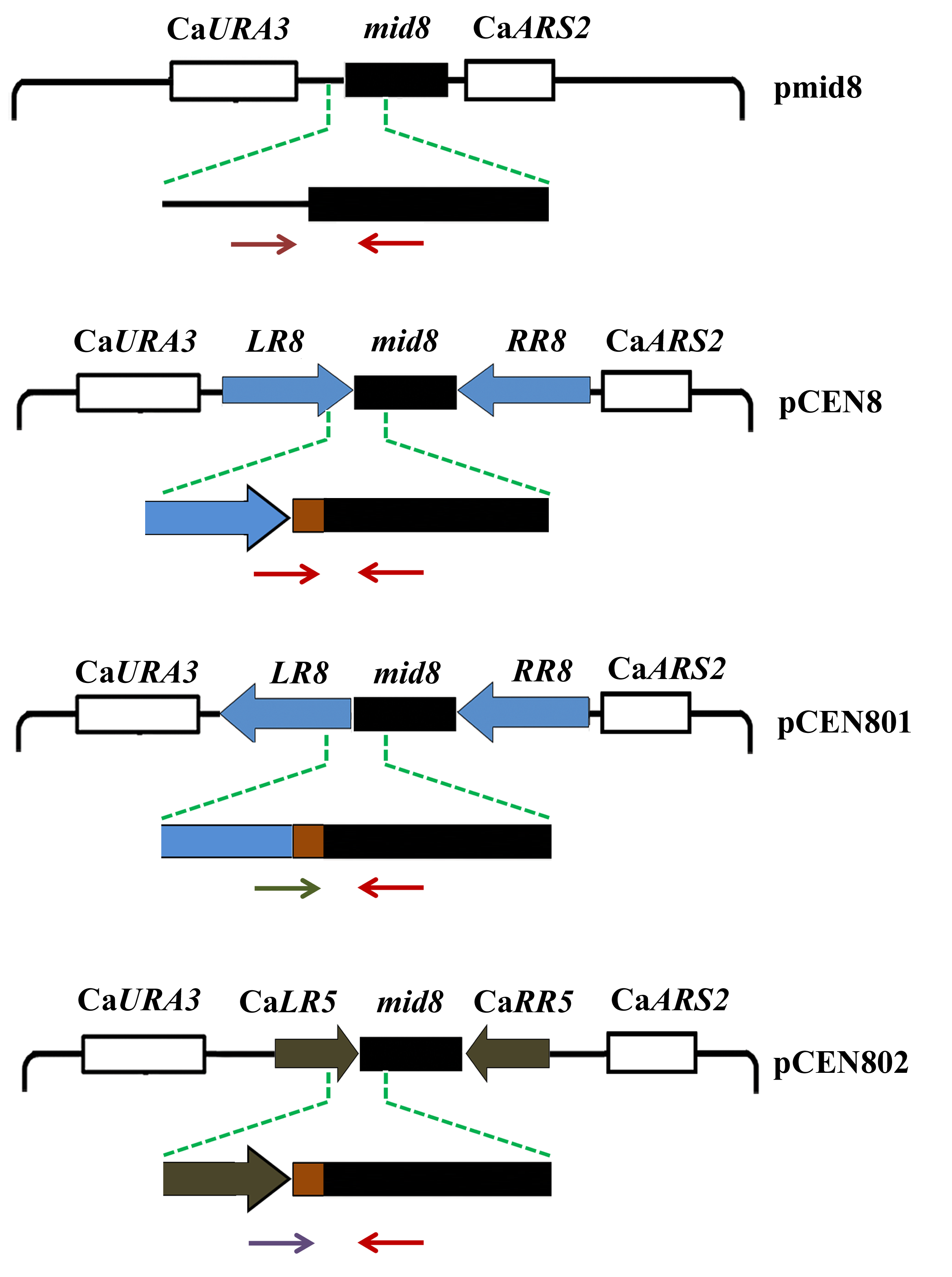

Supplement: S8 Fig — Schematics represent locations of plasmid specific primer-pairs for each plasmid used in the mitotic stability assays. The brown color demarcates a unique 6-bp SalI site in a plasmid, which is absent at the native locus. The specificity of the amplicon carrying plasmid-borne CEN8 was achieved by the addition of a unique engineered SalI site at the 3’ end of each primer. Schematics were not drawn to scale. (TIF) [file pgen.1005839.s009.tif]
