# Supplementary material for: Repeat-Associated Fission Yeast-Like Regional Centromeres in the Ascomycetous Budding Yeast Candida tropicalis
Source: PLoS Genet. 2016 Feb 4;12(2):e1005839. doi: 10.1371/journal.pgen.1005839 (PMC4741521; doi:10.1371/journal.pgen.1005839)
Supplement: S1 Table — (DOCX) [file pgen.1005839.s010.docx]

**S1 Table. The length and coordinates of the CENP-A and CENP-C binding as identified by the ChIP-seq analysis within an ORF-free region in *C. tropicalis*.** ORFs (>150 amino acids) flanking the centromeric regions in *C. tropicalis* were also mentioned.

| **Supercontig** | **Left-ORF coordinates** | **CENP-A enrichment coordinates** | **CENP-C enrichment coordinates** | **Right-ORF coordinates** |
| --- | --- | --- | --- | --- |
| Scnt 1 | CTRG_00438.3  943161-945461  (2301 bp) | 949076-951743  (2667 bp) | 948865-951786  (2921 bp) | CTRG_00441.3  956487-957638  (1152 bp) |
| Scnt 3 | CTRG_02628.3  1299395-1301008  (1614 bp) | 1305879-1308759  (2880 bp) | 1306051-1308808  (2757 bp) | CTRG_02630.3  1313510-1315759  (2250 bp) |
| Scnt 4 | CTRG_03206.3  417525-418517  (993 bp) | 422287-425399  (3112 bp) | 422382-425489  (3107 bp) | CTRG_03209.3  432424-436980  (4557 bp) |
| Scnt 5 | CTRG_04022.3  716396-718621  (2226 bp) | 722588-725374  (2786 bp) | 722611-725370  (2759 bp) | CTRG_04024.3  729276-732869  (3594 bp) |
| Scnt 7 | CTRG_05085.3  593445-594515  (1071 bp) | 609317-612223  (2906 bp) | 609308-612375  (3067 bp) | CTRG_05092.3  618891-622277  (3387 bp) |
| Scnt 8 | CTRG_05492.3  624683-627202  (2520 bp) | 633078-635757  (2679 bp) | 632961-635743  (2782 bp) | CTRG_05498.3  641514-646013  (4500 bp) |
| Scnt 9 | CTRG_05759.3  454183-458574  (4392 bp) | 463489-465628  (2139 bp) | 463418-465903  (2485 bp) | CTRG_05761.3  469773-470282  (510 bp) |
