# Supplementary material for: Repeat-Associated Fission Yeast-Like Regional Centromeres in the Ascomycetous Budding Yeast Candida tropicalis
Source: PLoS Genet. 2016 Feb 4;12(2):e1005839. doi: 10.1371/journal.pgen.1005839 (PMC4741521; doi:10.1371/journal.pgen.1005839)
Supplement: S2 Table — (DOCX) [file pgen.1005839.s011.docx]

**S2 Table. The length and coordinates of the inverted repeats (*IR*s) along with *mid* core region of each centromere in *C. tropicalis*.**

| **Supercontig** | **Left-repeat coordinates (*LR*)** | **Middle region coordinates (*mid*)** | **Right-repeat coordinates (*RR*)** |
| --- | --- | --- | --- |
| Scnt 1 | 945556-949426  (3870 bp) | 949427-951673  (2246 bp) | 951674-955555  (3881 bp) |
| Scnt 3 | 1301985-1304449  (2464 bp) | 1304450-1309419  (4969 bp) | 1309420-1311895  (2475 bp) |
| Scnt 4 | 418869-422567  (3698 bp) | 422568-425206  (2638 bp) | 425207-428971  (3764 bp) |
| Scnt 5 | 718785-722603  (3818 bp) | 722604-725145  (2541 bp) | 725146-728897  (3751 bp) |
| Scnt 7 | 595508-601206  (5698 bp) | 601207-612161  (10954 bp) | 612162-618031  (5869 bp) |
| Scnt 8 | 629391-633259  (3868 bp) | 633260-635891  (2631 bp) | 635892-639685  (3793 bp) |
| Scnt 9 | 459203-461413  (2210 bp) | 461414-466865  (5451 bp) | 466866-469025  (2159 bp) |
