# Supplementary material for: Repeat-Associated Fission Yeast-Like Regional Centromeres in the Ascomycetous Budding Yeast Candida tropicalis
Source: PLoS Genet. 2016 Feb 4;12(2):e1005839. doi: 10.1371/journal.pgen.1005839 (PMC4741521; doi:10.1371/journal.pgen.1005839)
Supplement: S3 Table — (DOCX) [file pgen.1005839.s012.docx]

**S3 Table. Tandem repeats (TR) within the *IR*s arms at the pericentric regions, using the Tandem Repeat Finder version 4.04.**

**Group 1:**

| Inverted repeat arm | Copy number | Repeat Consensus Sequence |
| --- | --- | --- |
| 1L | 11.4 | TTGATATGCTCGGCCGAAATGTAT |
| 1R | 11.4 | TTGTTATGCTCGGTGAAATGTAT |
| 3L | 8.6 | TTTATTTGATATGCTCGGCTCAATA |
| 3R | 11.3 | TGTTATGCTCGGCTGATGTTATT |
| 4L | 11.4 | TGTTATGCTCGGCTGAATGTTATT |
| 4R | 11.4 | TGTTATGCTCGGCTGAATGTTATT |
| 5L | 15.8 | TTTATGTTATGCTCGGCTGAAGTA |
| 5R | 10.5 | TTTTGTTATGCTCGGGTGAAGTA |
| 7L | 6.5 | TTGATATGCTCGGCCCAAATCTAAT |
| 7R | 6.5 | TTGATATGCTCGGCCCAAATCTAAT |
| 8L | 9.3 | TGTTATGCTCGGGTGAATTTATAT |
| 8R | 9.2 | TCGGGTGAGTTTATTTGTTATGC |
| 9L | 10.2 | TCGGGTGAGTTTATTTGTTATGC |
| 9R | 10.2 | TCGGGTGAGTTTATTTGTTATGC |

**(8L is followed by, and 8R preceded by, a slightly different 24bp TR with copy number 2-3)**

**Group 2:**

| Inverted repeat arm | Copy number | Repeat Consensus Sequence |
| --- | --- | --- |
| 1L | 7.4 | TGTCGCATCTATCA |
| 1R | 9.4 | TGTCGCATCAACCA |
| 3L | 7.6 | CATGTCGCATCTAC |
| 3R | 7.6 | CATGTCGCATCTAC |
| 4L | 2.4 | TGTCGCATCAACCA |
| 4R | 8.4 | TGTCGCATCTACCA |
| 5L | 7.4 | TGTCGCATCAACCA |
| 5R | 4.4 | TGTCGCATCTATCA |
| 7L | 4.4 | TGTCGCATCTATCA |
| 7R | 10.4 | TGTCGCATCTATCA |
| 8L | 7.6 | CATGTCGCATATAC |
| 8R | 7.6 | CATGTCGCATCAAC |
| 9L | 7.4 | TGTCGCATCTATCA |
| 9R | 3.4 | TGTCGCATCAACCA |

**Group 3:**

| Inverted repeat arm | Copy number | Repeat Consensus Sequence |
| --- | --- | --- |
| 1L | 24.9 | ATTATTTGATATGCTG |
| 1R | 22.9 | ATTATTTGTTATGCTG |
| 3L | 24.9 | TTTATTTGTTATGCTA |
| 3R | 22.9 | ATTATTTGATATGCTG |
| 4L | 27.9 | ATTATTTGATATGCTG |
| 4R | 26.9 | ATTATTTGATATGCTG |
| 5L | 26.9 | ATTATTTGATATGCTG |
| 5R | 26.9 | ATTATTTGTTATGCTG |
| 7L | 20.7 | TTTGATATGCTGATTA |
| 7R | 25.7 | TTTGATATGCTGATTA |
| 8L | 22.9 | TTTGATATGCTGTTTA |
| 8R | 19.7 | TTTGATATGCTGATTA |
| 9L, 9R | Missing | ------ |
