# Supplementary material for: Repeat-Associated Fission Yeast-Like Regional Centromeres in the Ascomycetous Budding Yeast Candida tropicalis
Source: PLoS Genet. 2016 Feb 4;12(2):e1005839. doi: 10.1371/journal.pgen.1005839 (PMC4741521; doi:10.1371/journal.pgen.1005839)
Supplement: S4 Table — (DOCX) [file pgen.1005839.s013.docx]

**S4 Table. Primers used in this study.**

| **Primer** | | **Amplification for** | | **Sequence (5’ to 3’)** | **Coordinates** | | **Size**  **(bp)** |
| --- | --- | --- | --- | --- | --- | --- | --- |
| **Ct*URA3* auxotroph strain (CtKS02)** | | | | | | | |
| KDOCt17 (F) | | *URA3* upstream sequence as *Xho*I and *Kpn*I fragment | | GGGGTACCGAAAAGATTTGGTTCGGATTATTAG | 3:123609-123633 | | 591 |
| KDOCt18 (R) | |  |  | CCGCTCGAGGTGTGTGTTTATGGATAGTAGTGAG | 3: 124176-124200 | |  |
| KDOCt19 (F) | | *URA3* downstream sequence as *Sac*I and *Sac*II fragment | | TCCCCGCGGCCGGTCAACTTGAATAGTTG | 3: 125004-125023 | | 713 |
| KDOCt20 (R) | |  |  | GGCCGAGCTCGTTGGTGTATTTGCAGGTTATGGG | 3: 125694-125717 | |  |
| **For CaGFP cloning** | | | |  |  | |  |
| SR67 | | CaGFP with actin terminator from pNIM1 plasmid as *SpeI* and *Xma*I fragment | | CGC ACTAGT ATG AGT AAG GGA GAA GAA CTT TTC AC |  | | 1125 |
| SR68 | |  |  | GGA CCCGGG CAT TTT ATG ATG GAA TGA ATG GGA TG |  | |  |
| **For CENP-A-GFP tagging** | | | | | | | |
| Cse4DS F | | CENP-A downstream sequence as *Cla*I and *Xho*I fragment | | GAC CTATCG ATA TTT GTA TAG AAG TAG TGT ACC ATATAG | 3:1334101-1334128 | | 499 |
| Cse4DS R | |  |  | CTT GCT CGA GATTACACGTGATTATGGGG | 3:1333629-1333647 | |  |
| Cse4 ORF F | | CENP-A ORF sequence as *Sac*I and *Spe*I fragment | | CCA GAG CTC GGTGATGTAGTTGAAACAGTTCG | 3:1334529-1334551 | | 419 |
| Cse4 ORF R | |  |  | GCA CTA GTTAGAATCCAGGACTGGCCC | 3:1334132-1334150 | |  |
| **For CENP-C-GFP tagging** | | | | | | | |
| KG113 | | Part of CENP-C ORF *Sac*II and *Spe*I fragment | | TCCCCGCGGAGACCACAAACACCAACTAGCG | 9: 475135-475157 | | 486 |
| KG119 | |  |  | GGA CTA GTC CAA GTA TCT TCA ACT TCT TCA GG | 9: 475598-475621 | |  |
| **For Nuf2-GFP tagging** | | | | | | | |
| KG127 | | Part of *NUF2* ORF as *Sac*II and *Spe*I fragment | | ATCACCGCGGGATTATAAACAGGAGAAAACCAATTTAGC | 8: 363551-363579 | | 695 |
| KG128 | |  |  | ATCAACTAGTTTTGATATTTTTGTTTAATTCTGTCATATATC | 8: 362884-362915 | |  |
| KG129 | | *NUF2* downstream sequences as *Apa*I and *Kpn*I fragment | | ATC GGG GCC CCA CTA ACC TGT ATA GAC CAA ATA ATT TG | 8: 362839-362866 | | 664 |
| KG1 | |  |  | GGGGTA CCA TTT TAT CAC CTT TGG GAA CAGG | 8: 362202-362225 | |  |
| **For Dad1-GFP tagging** | | | | | | | |
| SR126 | | Part of *DAD1* ORF as *Sac*II and *Spe*I fragment | | TCCCCGCGGCATCAGACATTCCTAATAGTACATCACC | 4: 1406945-1406972 | | 286 |
| SR127 | |  |  | GGACTAGTTTCATTTGGTTCGTCTGCTATATC | 4: 1407208-1407231 | |  |
| **For Ct*GAL1* Pr. Cloning** | | | | | | | |
| SR145.2 | | Ct*GAL1* promoter region as *Cla*I and *Sal*I fragment | | CTG CAT CGA TTGATGGTATAAATGATTAAATGTGTTTTTG | 6: 770491-770520 | | 1163 |
| SR146 | |  |  | GGT CGT CGA CAG TGT TTA TTA CTA TGA ACA AAA TCA GAT C | 6: 769358-769387 | |  |
| **CENP-A conditional mutant** | | | | | | | |
| VR37 | | CENP-A downstream sequence as *Xho*I and *Kpn*I fragment | | GGGGTACCGGTTTCTGGTGAAGAATG | 3:1333646-1333663 | | 399 |
| VR38 | |  |  | CCGCTCGAGTCAATCGAATGACAGAGG | 3:1334028-1334045 | |  |
| VR39 | | CENP-A upstream sequence as *Sac*I and *Sac*II fragment | | TCCCCGCGGGTTCTTGATCCAATGGTCTG | 3:1334898-1334917 | | 446 |
| VR40 | |  |  | CGAGCTCGGTACTGAATGGTTAGGTTC | 3:1335325-1335344 | |  |
| VR50 | | Part of CENP-A ORF as *Xho*I and *Kpn*I fragment | | CCG CTC GAG ATG GCC AAA CCG TCA GAA C | 3:1334827-1334845 | | 395 |
| VR51 | |  |  | CGG GGT ACC AGC TGT CCT CAC ACT CAT GC | 3:1334450-1334469 | |  |
| **CENP-C conditional mutant** | | | | | | | |
| VR41 | | CENP-C upstream sequence as *Kpn*I and *Xho*I fragment | | GGGGTACCGGTTTGGTCATTGGGTATCA | 9: 473610-473629 | | 428 |
| VR42 | |  |  | CCGCTCGAGATATCACTCCGTGGACTTTC | 9: 474019-474038 | |  |
| VR43 | | CENP-C downstream sequence as *Sac*I and *Sac*II fragment | | TCCCCGCGGATTTATGTACTTCACCATCATG | 9: 475725-475746 | | 371 |
| VR44 | |  |  | CGAGCTCCT AAT GAA GAT CCT GAA ACTG | 9: 476076-476096 | |  |
| VR52 | | Part of CENP-C ORF as *Xho*I and *Kpn*I fragment | | CCG CTC GAG ATG TAT TTG GCA AAT CTT GG | 9: 474053-474072 | | 642 |
| VR53 | |  |  | CGG GGT ACC ATC TGT AAA TGA TGA CGG TAG TC | 9: 474672-474695 | |  |
| ***NUF2* conditional mutant** | | | | | | | |
| KG1 | | *NUF2* downstream sequence as *Xho*I and *Kpn*I fragment | | GGGGTA CCA TTT TAT CAC CTT TGG GAA CAGG | 8: 362202-362225 | | 665 |
| KG2 | |  |  | CCG CTC GAG CACTAACCTGTATAGACCAAATAATTTG | 8: 362839-362866 | |  |
| KG3 | | *NUF2* upstream sequence as *Sac*I and *Sac*II fragment | | CGAGCTCCGTAGAGTTAGACCAAGTTTATTAACAC | 8: 364999-365026 | | 539 |
| KG4 | |  |  | TCCCCGCGGCCGAATTAGACACTGCTACTAC | 8: 364488-364512 | |  |
| KG84 | | *NUF2* ORF with a N terminus V5 tag as a *Sal*I and *Kpn*I fragment | | ACGCGTCGACATGGGTAAGCCTATCCCTAACCCTCTCCTCGGTCTCGATTCTACGTCTCGACTTAGCATAATTGGTG | 8: 364335-364356 | | 455 |
| KG76 | |  |  | GGGGTACCATTAATGTAAAATCATTAATTCCTCAGGC | 8: 363903-363933 | |  |
| ***DAD1* conditional mutant** | | | | | | | |
| SR25 | | *DAD1* upstream sequence as *Sac*I and *Sac*II fragment | | CCA GAG CTC GAC CTT CAT TTA CTT CAC CAC | 4:1406425-1406445 | | 509 |
| SR26 | |  |  | TCC CCG CGG GAT TGA TAA TGG GAG TAA ATA AAG AAT ATC TAA GG | 4:1406900-1406934 | |  |
| SR27 | | *DAD1* downstream sequence as *Xho*I and *Kpn*I fragment | | GCC GCT CGA GGT ACT ATC AAA ACT AAG CGA CTT TTC TTC | 4:1407335-1407363 | | 476 |
| SR28 | |  |  | GGG GTA CCG TTT GGA AGC TGC TGG TAT TG | 4:1407791-1407811 | |  |
| SR71 | | *DAD1* upstream sequence as *Bam*HI and *Pst*I fragment | | CGC GGA TCC CCA AAC TGC CAT TCT ATG ACC TG | 4:1406141-1406163 | | 473 |
| SR72 | |  |  | GCC CTG CAG GGA GAA GAT GAA GAA GAT GATGAT ATT G | 4:1406587-1406614 | |  |
| SR73 | | *DAD1* ORF with downstream sequence as *Sal*I and *Kpn*I fragment | | GAC CGT CGA CAT GAC ATC TAC ATC AGA CAT TCC | 4:1406935-1406957 | | 582 |
| SR74 | |  |  | CGT GGT ACC GTC CAT CTA TTG TCC ACC AC | 4:1407498-1407517 | |  |
| **For CENP-C-TAP tagging** | | | | | | | |
| CtCENP-C I (F) | | CENP-C ORF with upstream sequences | | CAA CAA AGA AAC GTA CTA CCAAGA G | 9: 475092–475116 | | 529 |
| CtMIF IV (R) | |  |  | ctt ttt cca tct tct ctt ttc CCA AGT ATC TTC AAC TTC TTC AGG | 9: 475598–475621 | |  |
| JNAT-3UTRF (F) | | CENP-C  downstream  sequences | | ACG AGG CAA GCT TGA TAT C GTA  GTG ATC GAT ATGTAA TAT ATA | 9: 475625- 475648 | | 702 |
| CtCENP-C II (R) | |  |  | GAT GAT GTT AGA TCT GCT TTA GAT AC | 9: 476303–476328 | |  |
| CtCENP-C III (F) | | *TAP* amplification | | CCT GAA GAA GTT GAA GAT ACT TGG GAA AAG AGA AGA TGG AAA AAG | 475598–475621 | | 777 |
| TAP-JNATR (R) | |  |  | GAT ACTGAT ACT AAC GCC GCC ATC CAG T GGA AGA GAT CCAGAT ATT GAA G |  | |  |
| TAP-JNATF (F) | | *NAT1* amplification | | CCT TCA ATA TCT GGA TCT  CTT CC ACT GGA TGG CGG  CGT TAG TAT C |  | | 1216 |
| JNAT-3UTRR (R) | |  |  | T TAT TATATT ACA TAT CGA TCA  CTA CGATAT CAA GCT TGC  CTC GTC CC |  | |  |
| **For Dad1-TAP tagging** | | | | | | | |
| DAD1 FP | | *DAD1* ORF with upstream sequences | | TTCAGCAGCTCTTGCCTTC | 4:1406675-1406693 | | 556 |
| DAD1 RP | |  |  | CTTTTTCCATCTTCTCTT TTC TTCATTTGGTTCGTCTGC | 4:1407214-1407231 | |  |
| DAD1-TAP FP | | *TAP*-Ca*URA3* amplification | | GCAGACGAACCAAAT GAA GAAAAGAGAAGATGGAAAAAG |  | | 2002 |
| DAD1_TAP RP | |  |  | GTCCATCTATTGTCC ACC ATACGACTCACTATAGGGCGAATTG |  | |  |
| DAD1-3UTR FP | | *DAD1* downstream sequences | | CAATTCGCCCTATAGTGAGTC GTA TGGTGGACAATAGATGGAC | 4: 1407499-1407517 | | 472 |
| DAD1-3UTR RP | |  |  | AGATTATGTTACGTTGCTTTAG | 4: 1407950-1407971 | |  |
| **For CENP-A-TAP tagging** | | | | | | | |
| CSE4 1F | | CENP-A ORF fragment | | GAACAGCTACTAGAGAGAGATAG | 3:1334703-1334725 | | 594 |
| CSE4 2R | |  |  | CTTTTTCCATCTTCTCTTTTCTAGAATCCAGGACTG | 3:1334132-1334146 | |  |
| CSE4-3UTR F | | CENP-A downstream sequences | | CAATTCGCCCTATAGTGAGTCGTAGTGTACCATATAGAATGTAAGAG | 3:1334091-1334116 | | 548 |
| CSE4 6R | |  |  | GTACCAATAGAGAATTCTAGG | 3:1333569-1333589 | |  |
| CSE4 3F | | TAP-Ca*URA3*  amplification | | CCAGTCCTGGATTCTAGAAAAGAGAAGATGGAAAAAG |  | | 2002 |
| CSE4-TAP R | |  |  | CTCTTACATTCTATATGGTACACTACGACTCACTATAGGGCGAATTG |  | |  |
| KG121 | | CENP-A along with *TAP* amplification | | ATG CGC GGC CGC GTG GGC ATC TAT CGA AAT CAG | 3:1334555-1334575 | | 1150 |
| KG78 | |  |  | GAC TAG TGG CCA ATT ATA AAT GTG AAG GGG G |  | |  |
| **For ChIP on Scnt 1** | | | | | | | |
| Cnt1FP (F) | |  | | GTA ATG GAA AAC TTA TCG | 1: 949444–949461 | | 299 |
| Cnt1RP (R) | |  | | ATA CCC ATG GAC TGT ACC | 1: 949726–949743 | |  |
| **For ChIP on Scnt 3** | | | | | | | |
| Cnt3-1FP (F) | |  | | CAG ATG GCC ATT GTA GTC | 3:1307083-1307100 | | 312 |
| Cnt3-2RP (R) | |  | | GTG GAC TTC AAT GGG AAT | 3:1307378-1307395 | |  |
| **For ChIP on Scnt 4** | | | | | | | |
| Cnt4-1FP (F) | |  | | AAG TTA TCC GAC CTA CAT | 4: 423744–423761 | | 153 |
| Cnt4-2RP (R) | |  | | GCA CTT GGT GAA TAT TCC | 4: 423880–423897 | |  |
| **For ChIP on Scnt 5** | | | | | | | |
| Cnt5-1FP (F) | |  | | CAC TAT TAC GGT TAC TAC | 5: 723504–723521 | | 190 |
| Cnt5-2RP (R) | |  | | ACT GGA TAA TTT CAG TAT | 5: 723677–723694 | |  |
| **For ChIP on Scnt 7** | | | | | | | |
| Cnt7-1FP (F) | |  | | ATT GAT GTG TTC TAC TAA | 7: 610333–610350 | | 313 |
| Cnt7-2RP (R) | |  | | GCT TCG GTA TTT CCT CTT | 7: 610629–610646 | |  |
| **For ChIP on Scnt 8** | | | | | | | |
| Cnt8-1FP (F) | |  | | AAG ATA TCC GAC ATA CAA | 8: 634434–634451 | | 153 |
| Cnt8-2RP (R) | |  | | TCG TTT TGT GAA CAT TCC | 8: 634570–634587 | |  |
| **For ChIP on Scnt 9** | | | | | | | |
| Cnt9-3FP (F) | |  | | GCG AGA AAA TTA GTG CGT | 9: 464926–464943 | | 135 |
| Cnt9-4RP (R) | |  | | GGG ATG TTC TGG GCA ATT | 9: 465044–465061 | |  |
| **For ChIP on Ct*LEU2*** | | | | | | | |
| Leu2-1 (F) | |  | | ATG TCC GTT AAA ACT AAA ACA ATT ACT G | 7: 165901–165928 | | 350 |
| Leu2-1RP (R) | |  | | TCA CTT GCA AAA TTA CAA GGT CTA A | 7: 166226–166250 | |  |
| **For qPCR on Scnt 8** | | | | | | | |
| RT7-CtCEN8-21 (F) | |  | | CCT TAA GTA AAG GTG ATG CAG AGG C | 8: 634535-634559 | | 95 |
| RT7-CtCEN8-21RP (R) | |  | | CTA AAG CTT TGA CCC ACT CA | 8: 634611-634630 | |  |
| RT9-CtCEN8-13 (F) | |  | | CAA TAT TTC ATC GTG TTT CAC CCG | 8: 629300-629323 | | 91 |
| RT9-CtCEN8-13RP (R) | |  | | AAC AAA TTA CCC CGA GAC TAA | 8: 629371-629391 | |  |
| RT10-CtCEN8FP (F) | |  | | ATT CAT GTT TGT GGG ATA AGA T | 8: 630440-630461 | | 89 |
| RT10-CtCEN8RP (R) | |  | | CAA TTG AAC TTG TGG ATA ACT CT | 8: 630507-630529 | |  |
| RT11-CtCEN8-15 (F) | |  | | TAT TAG CTA CCA CGC GAC ATA TGC | 8: 631360-631383 | | 103 |
| RT11-CtCEN8-15RP (R) | |  | | AGA ATT TTA CGA CAT GGA AA | 8: 631444-631463 | |  |
| RT12-CtCEN8-17 (F) | |  | | TTA TGC TGC GAA ACA CTT TGA TAT G | 8: 632206-632230 | | 95 |
| RT12-CtCEN8-17RP (R) | |  | | TCA TAC CAT AAA TGG AAC CAG | 8: 632281-632301 | |  |
| RT13-CtCEN8FP (F) | |  | | CCC TAC TAT TAG TTG CTC TGC TA | 8 :633511-633533 | | 91 |
| RT13-CtCEN8RP (R) | |  | | ATA AGG TCA TTC ATT TTG TTC TG | 8: 633580-633602 | |  |
| RT15-CtCEN8FP (F) | |  | | ATC ACA AGT CTC TTT GAT AGC TG | 8: 639646-639668 | | 91 |
| RT15-CtCEN8RP (R) | |  | | CAC GTT ACC TAT TTT CAA CTT TT | 8: 639715-639737 | |  |
| RT16-CtCEN8FP (F) | |  | | GTG AAA TTT TGT GTC CTA CGC | 8: 635491-635511 | | 95 |
| RT16-CtCEN8RP (R) | |  | | ATT GAT GTT GAA AAG TTT GGA | 8: 635565-635585 | |  |
| RT17-CtCEN8FP (F) | |  | | TCC CTT ATG TAT GGT CAG TTC ACC C | 8: 636079-636103 | | 122 |
| RT17-CtCEN8RP (R) | |  | | GGT AAT TTG CTC CTG TAT TTT CGG CT | 8: 636174-636200 | |  |
| **For qPCR on native *CEN8*** | | | | | | | |
| SalI CC only FP |  | | ACG CGT CGA CGT AGT TAT CTA GAT GCA ATT TGT TTG | | 8:633331- 633356 |  | |
| RT-pCtCENRP |  | | TAT TAC CTA CAA ATA ACT TCA TCA AGT C | | 8: 633382-633409 | 78 | |
| **For qPCR on pCEN8** | | | | | | | |
| pCEN8ChIP FP |  | | AAC CTT GAT TCA AAG TAT TGT GTC GAC | | 8:633312-633332 |  | |
| RT-pCtCENRP |  | | TAT TAC CTA CAA ATA ACT TCA TCA AGT C | | 8: 633382-633409 | 97 | |
| **For qPCR on pmid8** | | | | | | | |
| RT- CC+IR and CC FP |  | | CTT GCA TGC CTG CAG GTC GAC | |  |  | |
| RT-pCtCEN RP |  | | TAT TAC CTA CAA ATA ACT TCA TCA AGT C | | 8: 633382-633409 | 100 | |
| **For qPCR on pCEN801** | | | | | | | |
| KG243 |  | | TGA AAC ACG ATG AAA TAT TGG TCG AC | |  |  | |
| RT-pCtCEN RP |  | | TAT TAC CTA CAA ATA ACT TCA TCA AGT C | | 8: 633382-633409 | 105 | |
| **For qPCR on pCEN802** | | | | | | | |
| KG242 |  | | AAAACACAACGTACTGGTCGAC | |  |  | |
| RT-pCtCEN RP |  | | TAT TAC CTA CAA ATA ACT TCA TCAAGT C | | 8: 633382-633409 | 101 | |
| **For qPCR on Ct*LEU2*** | | | | | | | |
| Leu2-3FP (F) | |  | | TAA AAA TCA TTT AAT TGG TGG TG | 7: 166032–166054 | |  |
| Leu2-3RP (R) | |  | | ACA GCA TCT GAT GAT TTA GCA CAT | 7: 166101–166124 | | 93 |
| **Sanger sequencing** | | | | | | | |
| **Scnt 1** | |  | |  |  | |  |
| Cnt1 FP | |  | | GTA ATG GAA AAC TTA TCG | 1: 949444-949461 | |  |
| Cnt1 seq2 RP | |  | | CGA GTG AAG TAC TGT CAA TCT C | 1: 950967-950988 | |  |
| Cnt1 seq3 RP | |  | | AGT ACG AAA TGA GAG CAA ATG TC | 1: 950190-950212 | |  |
| Cnt1 seq4 RP | |  | | CTC AGC GTA CAC GTT TTC AC | 1: 952682-952698 | |  |
| Cnt1 seq5 RP | |  | | ACA TTA ATG ACC TTC ACT GCC AG | 1: 950840-950862 | |  |
| **Scnt 9** | |  | |  |  | |  |
| Cnt9 seq FP | |  | | ATG GGG TAT GAT GCT TAT TT | 9: 461384-461403 | |  |
| Cnt9 seq RP | |  | | GAT CTA CTT CAT CTA CAA TT | 9: 462884-462903 | |  |
| Cnt9 seq2 FP | |  | | CCA AGG TTC AGG ACT CCA AG | 9: 458541-458560 | |  |
| Cnt9 seq2 RP | |  | | GGA GAG AAA AGG TAG CCC AG | 9: 463284–463303 | |  |
| **Scnt 7** | |  | |  |  | |  |
| Cnt7 seq FP | |  | | CAA GCC TTC AGA GAA AGA GAC AC | 7: 602766-602788 | |  |
| Cnt7 seq FP | |  | | GTT CGT TCG TTT TTC ACT GTT G | 7: 603199-603230 | |  |
| **For CHEF (Southern blot)** | | | | | | | |
| **Scnt 1** | |  | |  |  | |  |
| CtCHEF-Cnt1-USFP (F) | |  | | CAG CAA AGG AAT TAT ATC TTG TTCT | 1: 943100-943124 | | 1079 |
| CtUS1-2 (R) | |  | | GCT TGT AAC AAG GTT GAA ATT GAC C | 1: 944155-944179 | |  |
| **Scnt 5** | |  | |  |  | |  |
| CtCHEF-Cnt5-USFP (F) | |  | | AGT TTG TGA ATC ATT AGT TTA GCT T | 5: 714937-714961 | | 932 |
| CtCHEF-Cnt5-USRP (R) | |  | | CAA GTC ATC GAT ACT ACT GAT AGT G | 5: 715845-715869 | |  |
| **Scnt 7** | |  | |  |  | |  |
| CtCHEF-Cnt7-USFP (F) | |  | | TAC ATT GTT GGA ATA AGT CAT AGT G | 7: 593348-593372 | | 986 |
| CtCHEF-Cnt7-USRP (R) | |  | | CCT ATA TTT CAG AAG TAC CAG AAA G | 7: 594310-594334 | |  |
| **Scnt 8** | |  | |  |  | |  |
| CtCEN8-3 (F) | |  | | CAT TCA TTT CGT TGT CTT GTT GAT | 8: 624420-624443 | | 1213 |
| CtUS8-2 (R) | |  | | AAG GAT CAA CTC ATT GAA TGC GTC | 8: 625610-625633 | |  |
| **Scnt 9** | |  | |  |  | |  |
| CtCHEF-Cnt9-USFP (F) | |  | | ATC ATG AGA ATA CAA AGA GAA AGT T | 9: 456368-456392 | | 945 |
| CtCHEF-Cnt9-USRP (R) | |  | | AAC TTT TGT AGT CTA TCC AAC TCT G | 9: 457337-457361 | |  |
| **For *mid core8* cloning** | | | | | | | |
| SalI CC only FP (F) | | *mid* core of Scnt 8 | | ACG CGT CGA CGT AGT TAT CTA GAT GCA ATT TGT TTG | 8:633331-633356 | | 2557 |
| BamHI CC only RP (R) | |  |  | CGG GAT CCG GTA ATT GTA GAT GAA GTA GAT CTA TG | 8: 635861-635887 | |  |
| **For *CEN8* cloning** | | | | | | | |
| SalI CConly FP (F) | | *mid* core with right repeat (*RR*) of Scnt 8 | | ACG CGT CGA CGT AGT TAT CTA GAT GCA ATT TGT TTG | 8: 633331-633356 | | 6334 |
| CEN8RP (R) | |  |  | CGG GAT CCG TTC CAA TCG TTG TCA CCA ATG TG | 8: 639672-639695 | |  |
| CEN8FP (F) | | left repeat (*LR*) of Scnt 8 | | TGC ACT GCA GCA ATA TTT CAT CGT GTT TCA CCC G | 8: 629300-629323 | | 4032 |
| SalI IRonly RP (R) | |  |  | ACG CGT CGA CAA TAC TTT GAA TCA AGG TTA GCA ATG | 8: 633305-633331 | |  |
| **For *LR8* cloning in direct orientation** | | | | | | | |
| KG235 | | left repeat (*LR*) of Scnt 8 | | ATG CGT CGA CCA ATA TTT CAT CGT GTT TCA CCC G | 8: 629300-629323 | | 4032 |
| KG236 | |  |  | CAG TCT GCA GAA TAC TTT GAA TCA AGG TTA GCA ATG | 8: 633305-633331 | |  |
| **For Ca*LR5* cloning** | | | | | | | |
| KG229 | | left repeat (*LR*) of Ca*CEN5* | | AGT CCT GCA GGC ATT CGA AGG ACA TTA ATT AAC G | Ca5: 466301-466324 | | 2254 |
| KG230 | |  |  | ATGCGTCGACCAGTACGTTGTGTTTTGAAGTCCTC | Ca5: 468530-468554 | |  |
| **For Ca*RR5* cloning** | | | | | | | |
| KG231 | | right repeat (*RR*) of Ca*CEN5* | | AGCGGATCCCTTTTTATTCCAGTATTCTGATTGATCTATTTATC | Ca5: 474066-474000 | | 2317 |
| KG232 | |  |  | ATGCGGATCCGATGTTGTTGTGGTAGCCATAGTGTG | Ca5: 471750-471775 | |  |
