# Supplementary material for: Repeat-Associated Fission Yeast-Like Regional Centromeres in the Ascomycetous Budding Yeast Candida tropicalis
Source: PLoS Genet. 2016 Feb 4;12(2):e1005839. doi: 10.1371/journal.pgen.1005839 (PMC4741521; doi:10.1371/journal.pgen.1005839)
Supplement: S5 Table — (DOCX) [file pgen.1005839.s014.docx]

**S5 Table. Strains used in this study.**

| **Yeast strains** | **Genotype** | **Reference** |
| --- | --- | --- |
| MYA-3404 | Clinical isolates (wild-type) | 37 |
| CtKS01 | *URA3*/*ura3*::*FRT* | This study |
| CtKS02 | *ura3::FRT*/*ura3*::*FRT* | This study |
| CtKS03 | *ura3::FRT/ura3::FRT HIS1/his1::FRT* | This study |
| CtKS04 | *ura3::FRT/ura3::FRT his1::FRT/his1::FRT* | This study |
| CtKS05 | *ura3::FRT/ura3::FRT his1::FRT/his1::FRT ARG4/arg4::FRT* | This study |
| CtKS06 | *ura3::FRT/ura3::FRT his1::FRT/his1::FRT arg4::FRT/arg4::FRT* | This study |
| CtKS100 | *ura3::FRT/ura3::FRT his1::FRT/his1::FRT CSE4/CSE4::CSE4-GFP (CaHIS1)* | This study |
| CtKS101 | *ura3::FRT/ura3::FRT his1::FRT/his1::FRT arg4::FRT/arg4::FRT CSE4/CSE4::CSE4-TAP (CaURA3)* | This study |
| CtKS102 | *ura3::FRT/ura3::FRT his1::FRT/his1::FRT arg4::FRT/arg4::FRT CSE4/CSE4::CSE4-TAP (CaHIS1)* | This study |
| CtKS103 | *ura3::FRT/ura3::FRT his1::FRT/his1::FRT cse4::FRT/CSE4* | This study |
| CtKS104 | *ura3::FRT/ura3::FRT his1::FRT/his1::FRT CSE4::FRT/CSE4::GAL1Pr.-CSE4 (CaURA3)* | This study |
| CtKS200 | *ura3::FRT/ura3::FRT his1::FRT/his1::FRT arg4::FRT/arg4::FRT MIF2/MIF2::MIF2-GFP (CaHIS1)* | This study |
| CtKS201 | *MIF2*/*MIF2*::*MIF2*-*TAP* (*NAT*^R^) | This study |
| CtKS202 | *ura3::FRT/ura3::FRT his1::FRT/his1::FRT MIF2/MIF2::GAL1Pr.-MIF2 (CaURA3)* | This study |
| CtKS203 | *ura3::FRT/ura3::FRT his1::FRT/his1::FRT mif2::FRT/MIF2::GAL1Pr.-MIF2 (CaURA3)* | This study |
| CtKS300 | *ura3::FRT/ura3::FRT his1::FRT/his1::FRT arg4::FRT/arg4::FRT NUF2/NUF2::NUF2-GFP (CaHIS1)* | This study |
| CtKS302 | *ura3::FRT/ura3::FRT his1::FRT/his1::FRTarg4::FRT/arg4::FRT nuf2::FRT/NUF2* | This study |
| CtKS303 | *ura3::FRT/ura3::FRT his1::FRT/his1::FRTarg4::FRT/arg4::FRT nuf2::FRT/NUF2::GAL1Pr.-V5-NUF2 (CaURA3)* | This study |
| **Yeast strains** | **Genotype** | **Reference** |
| CtKS400 | *ura3::FRT/ura3::FRT his1::FRT/his1::FRT DAD1/DAD1::DAD1-GFP (CaHIS1)* | This study |
| CtKS401 | *ura3::FRT/ura3::FRT his1::FRT/his1::FRT DAD1/DAD1::DAD1-TAP (CaURA3)* | This study |
| CtKS402 | *ura3::FRT/ura3::FRT his1::FRT/his1::FRT dad1::FRT/DAD1* | This study |
| CtKS403 | *ura3::FRT/ura3::FRT his1::FRT/his1::FRT dad1::FRT/DAD1::GAL1Pr.-DAD1 (CaURA3)* | This study |
